# Combined proteomic and biochemical analyses redefine the consensus sequence requirement for epidermal growth factor-like domain hydroxylation

**DOI:** 10.1016/j.jbc.2022.102129

**Published:** 2022-06-11

**Authors:** Lennart Brewitz, Bruce C. Onisko, Christopher J. Schofield

**Affiliations:** 1 Chemistry Research Laboratory, Department of Chemistry and the Ineos Oxford Institute for Antimicrobial Research, University of Oxford, Oxford, United Kingdom; 2 OniPro LLC, Kensington, California, USA

**Keywords:** epidermal growth factor-like domain, protein hydroxylation, aspartate/asparagine-β-hydroxylase, 2-oxoglutarate, α-ketoglutarate oxygenase, fibulin, fibrillin, latent-transforming growth factor β-binding protein, proteomics, 2OG, 2-oxoglutarate, AspH, aspartate/asparagine-β-hydroxylase, EGFD, epidermal growth factor-like domain, FAS, ammonium iron(II) sulfate hexahydrate, hFX, human coagulation factor X, LAA, L-ascorbic acid, MS, mass spectrometry, PTM, post-translational modification, SPE-MS, solid phase extraction coupled to MS, SPPS, solid phase peptide synthesis, TPR, tetratricopeptide repeat

## Abstract

Epidermal growth factor-like domains (EGFDs) have important functions in cell–cell signaling. Both secreted and cell surface human EGFDs are subject to extensive modifications, including aspartate and asparagine residue C3-hydroxylations catalyzed by the 2-oxoglutarate oxygenase aspartate/asparagine-β-hydroxylase (AspH). Although genetic studies show AspH is important in human biology, studies on its physiological roles have been limited by incomplete knowledge of its substrates. Here, we redefine the consensus sequence requirements for AspH-catalyzed EGFD hydroxylation based on combined analysis of proteomic mass spectrometric data and mass spectrometry–based assays with isolated AspH and peptide substrates. We provide cellular and biochemical evidence that the preferred site of EGFD hydroxylation is embedded within a disulfide-bridged macrocycle formed of 10 amino acid residues. This definition enabled the identification of previously unassigned hydroxylation sites in three EGFDs of human fibulins as AspH substrates. A non-EGFD containing protein, lymphocyte antigen-6/plasminogen activator urokinase receptor domain containing protein 6B (LYPD6B) was shown to be a substrate for isolated AspH, but we did not observe evidence for LYPD6B hydroxylation in cells. AspH-catalyzed hydroxylation of fibulins is of particular interest given their important roles in extracellular matrix dynamics. In conclusion, these results lead to a revision of the consensus substrate requirements for AspH and expand the range of observed and potential AspH-catalyzed hydroxylation in cells, which will enable future study of the biological roles of AspH.

Epidermal growth factor-like domains (EGFDs) are common structural elements in secreted and cell surface–bound human proteins and are also present in the extracellular domains of transmembrane proteins, where they can be present as isolated domains or as an array of multiple (tandem) domains (1, 2, 3). EGFDs are typically composed of 30 to 50 aa residues and have a conserved fold comprising a two-stranded antiparallel β-sheet, but manifest low overall sequence similarity (1, 2, 3). The EGFD fold is stabilized by three intradomain disulfide linkages; most NMR and crystal structures of EGFDs manifest a C1–C3, C2–C4, and C5–C6 disulfide pattern (Fig. 1) (4, 5, 6, 7, 8). EGFDs have important functions including in cell–cell signaling, blood clotting, and extracellular matrix formation, as evidenced by clinically observed mutations to EGFDs. Mutations in genes encoding for EGFDs of fibrillin-1 are associated with Marfan syndrome, a connective tissue disorder (9), and mutations in EGFDs of factor IX are associated with hemophilia B, a blood clotting disease (10).

**Figure 1:**
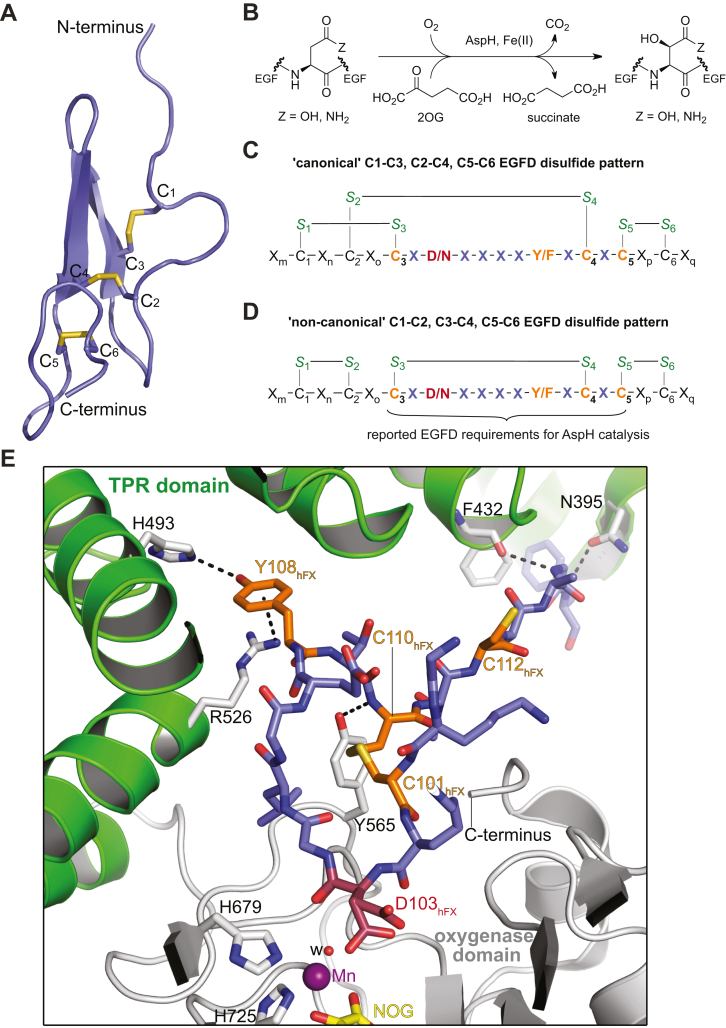
**AspH-catalyzed EGFD hydroxylation.***A*, view from a crystal structure of human coagulation factor IX (hFIX) EGFD1 (PDB ID: 1EDM (70)). The fold of hFIX contains a two-stranded antiparallel β-sheet and a ‘canonical’ C1–C3, C2–C4, and C5–C6 disulfide pattern, which is characteristic of most EGFD folds. Cystine sulfurs are in *yellow* and are numbered from the N to the C terminus. *B*, the AspH reaction. *C* and *D*, schematic depiction of an EGFD bearing (*C*) the ‘canonical’ C1–C3, C2–C4, and C5–C6 disulfide pattern, which is not a substrate for AspH (41), and (*D*) a ‘non-canonical’ C1–C2, C3–C4, and C5–C6 disulfide pattern, which is a substrate for AspH (41). The sites of AspH-catalyzed EGFD hydroxylation are in *red*; EGFD residues which define the currently accepted consensus sequence for AspH-catalyzed EGFD hydroxylation (10, 29, 46, 56, 57, 58) are in *orange* and *lavender*; cystine sulfurs are in *green*; numbered cysteine residues indicate their relative position in the consensus EGFD sequence; X denotes an apparently random proteinogenic amino acid and X_m–q_ denote a combination of (multiple) apparently random proteinogenic amino acids. *E*, view from an AspH structure in complex with a human coagulation factor X (hFX) EGFD1-derived peptide showing direct interactions of the C101_hFX_-C110_hFX_ bridged peptide with R526, H493, F432, and N395 of the AspH TPR domain (PDB ID: 5JZ8 (41)). Mn: manganese, an inactive substitute for Fe(II); w: water. The AspH TPR domain is a *green cartoon* and the AspH catalytic oxygenase domain is a *gray cartoon*. The hFX peptide is in *slate blue*, the site of hFX hydroxylation (D103_hFX_) is in *red*, and conserved residues reported to be required for AspH-catalyzed EGFD hydroxylation are in *orange*. *N*-oxalylglycine (NOG) is an inactive substitute for the natural cosubstrate 2OG (71) and is in *yellow*. AspH, aspartate/asparagine-β-hydroxylase; EGFD, epidermal growth factor-like domain; PDB, Protein Data Bank; TPR, tetratricopeptide repeat.

EGFDs are subject to extensive post-translational modifications (PTMs), some of which alter their chemical properties and which are reported to regulate *inter alia* protein secretion and function (11, 12, 13). Enzyme-catalyzed EGFD PTMs include *N*-linked asparagine residue glycosylation (14, 15, 16), serine and threonine residue *O*-glycosylation (*O*-glycosylated EGFD serine and threonine residues can be substrates for further enzyme-catalyzed glycosylation reactions) (12, 13, 17, 18, 19), and aspartate and asparagine residue hydroxylation (20, 21, 22). The effect of EGFD serine and threonine residue *O*-glycosylation on notch signaling has been extensively studied, in part because mutations leading to catalytically inactive human glycosyltransferases are associated with hereditary diseases, for example, Dowling–Degos disease and Adams–Oliver syndrome (23, 24, 25, 26, 27).

By contrast to EGFD serine and threonine residue *O*-glycosylation, the biological function of EGFD aspartate and asparagine residue hydroxylation is less well understood (28). Stereospecific β-hydroxylation of EGFD aspartate and asparagine residues is catalyzed by the 2-oxoglutarate (2OG) and Fe(II)-dependent aspartate/asparagine-β-hydroxylase (AspH, Fig. 1*B*) (29, 30, 31), which belongs to the 2OG oxygenase structural superfamily, members of which have important physiological roles in humans, including in lipid metabolism (32, 33), collagen biosynthesis (34, 35), nucleic acid repair (36, 37), hypoxia sensing (38, 39), and chromatin modification (36, 40).

AspH is highly unusual amongst 2OG-dependent hydroxylases in that it has only two Fe(II)-binding ligands (41, 42, 43). Mutations in the catalytic domain of human AspH, which likely reduce its catalytic activity and may thus indirectly alter levels of EGFD hydroxylation, are associated with developmental defects (Traboulsi syndrome) (44, 45, 46, 47, 48). AspH levels in cells are regulated by hypoxia, and AspH catalysis has the potential to be regulated by oxygen availability (49, 50, 51, 52). In some types of invasive cancers, AspH is upregulated on the cell surface and might indirectly alter levels of EGFD hydroxylation, thus rendering AspH a proposed target for cancer treatment (53, 54, 55).

It appears that AspH catalyzes the hydroxylation of those aspartate and asparagine residues in EGFDs, which are embedded in a C_3_-X-D/N-X-X-X-X-F/Y-X-C_4_-X-C_5_ consensus motif (Fig. 1) (10, 29, 46, 56, 57, 58). However, the presence of this consensus sequence does not inform on the degree of EGFD hydroxylation in humans, which varies substantially, from apparently none to apparently complete hydroxylation. Of the >100 human EGFDs that match the criterion for potential AspH-catalyzed hydroxylation, only some of those present in *inter alia* coagulation factors (20, 21, 22) and related vitamin K-dependent enzymes (59), notch (60), fibrillins (61, 62), fibulins (63), uromodulin (64), thrombomodulin (57, 65), complement proteases (64, 66, 67), latent-transforming growth factor β-binding proteins (62, 68), and low-density lipoprotein receptors (57) have been reported to be (partially) hydroxylated. Considering the proposed role of AspH upregulation in cancer progression (53, 54, 55), the identification of AspH substrates is important to inform on downstream signaling pathways, which may help to rationalize phenotypes associated with modulation of AspH levels or activity.

Recently, we reported structural and biochemical evidence that the EGFD disulfide connectivity has a profound impact on AspH catalysis (41). Despite bearing the EGFD consensus sequence for AspH-catalyzed hydroxylation, neither a reduced linear EGFD nor the corresponding oxidized EGFD with the canonical disulfide pattern (*i.e.*, with C1–C3, C2–C4, and C5–C6 disulfide linkages, Fig. 1*C*) were found to be substrates for isolated recombinant human AspH. By contrast, a corresponding noncanonical EGFD disulfide isomer, in which EGFD C1–C2, C3–C4, and C5–C6 linked disulfides are present (Fig. 1*D*), was hydroxylated by AspH (41). AspH–substrate complex crystal structures reveal that EGFD residues embedded in the C3–C4 macrocycle directly interact with both the catalytic domain and the adjacent tetratricopeptide repeat (TPR) domain (41). The side chain of the F/Y residue that is part of the EGFD consensus motif required for hydroxylation, binds in a hydrophobic pocket located between TPR repeats 5 and 6 (Fig. 1*E*)—note that the substitution of a tyrosine residue at this position with a valine residue has been reported to result in reduced levels of AspH-catalyzed EGFD hydroxylation (69). Interactions of EGFD residues with the TPR domain remote from the AspH active site have also been observed (41).

Here, we report cellular and biochemical evidence that macrocyclic EGFD disulfide isomers in which the hydroxylation site is embedded in a disulfide-bridged macrocycle composed of 10 amino acid residues are preferred substrates for human AspH. The results support previous work showing EGFDs as AspH substrates but lead to a revision of the EGFD consensus sequence requirements for AspH-catalyzed hydroxylation. They also reveal the potential of AspH to catalyze hydroxylations of non-EGFD–containing proteins. Importantly, EGFDs in human fibulins, for which EGFD hydroxylation would not have been anticipated based on the currently accepted criteria for EGFD hydroxylation, are shown to be hydroxylated by AspH.

## Results

### EGFD disulfide requirements for productive AspH catalysis

Previously, we have shown that recombinant human AspH does not catalyze the hydroxylation of linear (acyclic) EGFD-derived peptides *in vitro* but requires the site of EGFD hydroxylation to be buried within a C3–C4 disulfide-bridged macrocycle (41). The latter contrasts with the C2–C4 disulfide-bridged macrocycle (41, 49), which is the disulfide connectivity that has been most commonly observed in reported EGFD structures (4, 5, 6, 7, 8) and which we did not find to be an AspH substrate (41). However, investigations on the reactivity of AspH with other macrocyclic EGFD disulfide isomers have not yet been reported. The isomerization of EGFD disulfides has been calculated to be energetically feasible (70) and the spatial arrangement of the six cysteine residues in reported AspH substrate EGFD structures allows, in principle, for formation of different types of intramolecular disulfide connectivity patterns (7, 8). Structures of EGFDs with unusual disulfide bond connectivities have also been reported, but these EGFDs do not bear the consensus sequence for AspH-catalyzed hydroxylation (71, 72, 73, 74). We therefore systematically investigated whether different EGFD disulfide isomers, that bury the identified site of AspH-catalyzed hydroxylation within the macrocycle, are AspH substrates.

**Figure 2:**
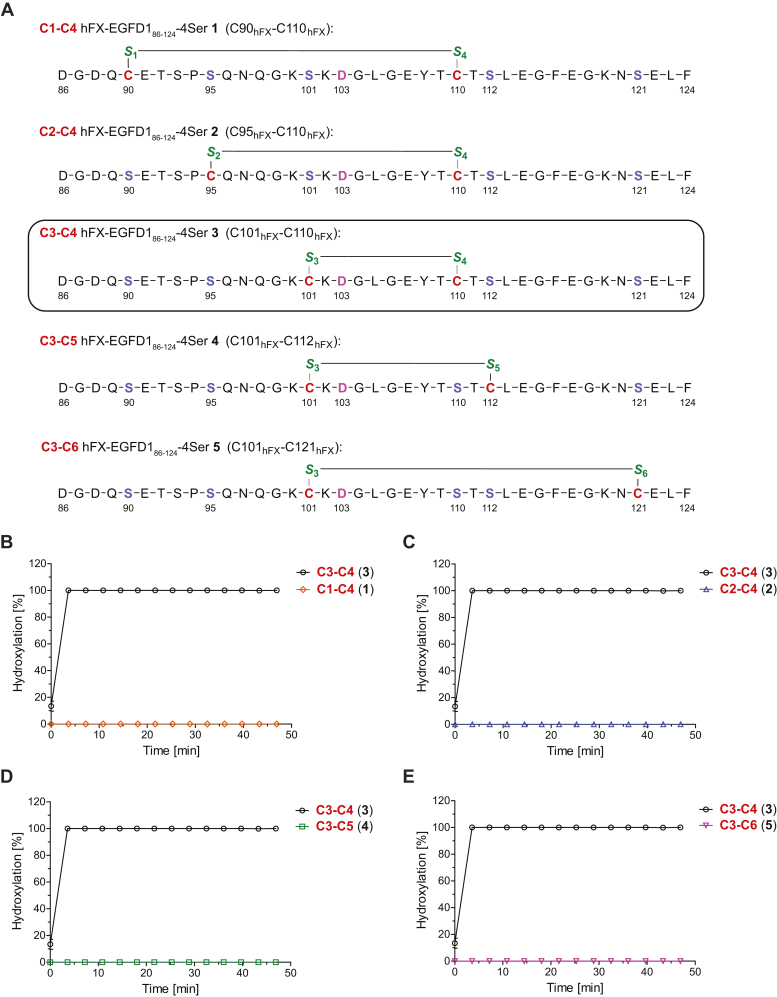
**A C3–C4 EGFD disulfide enables productive AspH catalysis.***A*, structures of the disulfide isomers hFX-EGFD1_86–124_-4Ser **1** to **5** used in this work; note that all the WT hFX EGFD1 cysteine residues except for the two in *red* were substituted for serine residues. The incubations of AspH with (*B*) the C1–C4 isomer **1** (*orange diamonds*), (*C*) the C2–C4 isomer **2** (*lavender triangles*), (*D*) the C3–C5 isomer **4** (*green boxes*), and (*E*) the C3–C6 isomer **5** (*pink inverse triangles*) of hFX-EGFD1_86–124_-4Ser were compared to the C3–C4 isomer **3** of hFX-EGFD1_86–124_-4Ser (*black circles*), which is a reported AspH substrate (41, 49). The results show that AspH only catalyzes hydroxylation of the C3–C4 hFX-EGFD1_86–124_-4Ser isomer **3**. The AspH hydroxylation site (D103_hFX_) is in *pink*; cystines are in *red*; cystine sulfurs are in *green*; serine residues substituting for hFX cysteines are in *lilac*; numbering is according to the sequence of hFX (UniProt ID: P00742). SPE-MS assays were performed as described in the Experimental procedures. Measurement times were normalized to the first sample injection analyzed after the addition of AspH to the substrate mixture (t = 0 s), by which time low levels of hydroxylation were manifest; results are means of three independent runs (n = 3; mean ± SD). AspH, aspartate/asparagine-β-hydroxylase; EGFD, epidermal growth factor-like domain; hFX, human coagulation factor X.

Four of the six EGFD1 cysteine residues of the validated AspH substrate human coagulation factor X (hFX), which is reported to be quantitatively hydroxylated at D103 *in vivo* (20, 21), were systematically substituted for serine residues to afford the isomerization-stable disulfide-bridged macrocycles hFX-EGFD1_86–124_-4Ser **1** to **5** (Fig. 2*A*). Note that only hFX-EGFD1_86–124_-4Ser disulfide isomers that bury the site of AspH-catalyzed hydroxylation within a macrocycle were synthesized because AspH does not accept linear substrates (41). The C3–C4 isomer of hFX-EGFD1_86–124_-4Ser (**3**) is a reported substrate for recombinant human AspH and has been used for crystallographic studies (41, 75, 76). The four possible disulfide isomers of hFX-EGFD1_86–124_-4Ser **3** that bury the site of AspH-catalyzed hydroxylation within the disulfide-bridged macrocycle, *i.e.*, C1–C4 hFX-EGFD1_86–124_-4Ser **1**, C2–C4 hFX-EGFD1_86–124_-4Ser **2**, C3–C5 hFX-EGFD1_86–124_-4Ser **4**, and C3–C6 hFX-EGFD1_86–124_-4Ser **5**, however, were not hydroxylated by N-terminally truncated recombinant human AspH (His_6_-AspH_315–758_) (41) under the tested reaction conditions (Fig. 2, *B*–*E*). The results reveal the importance of the C3–C4 EGFD disulfide for productive AspH catalysis and suggest that disulfide-bridged 10 amino acid membered macrocycles are preferred AspH substrates.

The currently accepted EGFD consensus sequence requirements for AspH catalysis imply that the presence of the EGFD C4-X-C5 three residue motif is necessary for AspH catalysis (Fig. 1, *C* and *D*) (10, 29, 46, 56, 57, 58). However, analysis of the results presented in Figure 2 led to the proposal that the position of EGFD C5 relative to C4 should not affect AspH catalysis. We therefore analyzed publicly available human proteomic data for aspartate/asparagine hydroxylation in EGFDs that do not contain the C4-X-C5 motif.

### Analysis of human proteomes

We analyzed available human proteomic data for aspartate and asparagine hydroxylation in EGFDs with a focus on those that do not bear the C4-X-C5 motif to test the proposals that the C3–C4 EGFD disulfide is required for productive AspH catalysis and that the relative position of C5 with respect to C4 does not affect AspH catalysis. We performed proteomic searches using the multinotch software MetaMorpheus (https://github.com/smith-chem-wisc/MetaMorpheus) (77) for potentially hydroxylated aspartate and asparagine residues, while also searching for hydroxylation/oxidation of glutamate, phenylalanine, histidine, lysine, methionine, proline, glutamine, arginine, tryptophan, and tyrosine residues. This approach has been found to improve the identification of hydroxylated proline residues by a factor of ∼9 when compared to use of the MaxQuant software (Max-Planck-Institute of Biochemistry; https://maxquant.org) (78). We chose to investigate data from female reproductive tissue as these generally contain high levels of AspH and EGFD-containing proteins that are putative AspH substrates (53, 79, 80, 81).

Analysis of human ovary-derived proteomic data (82) provides clear evidence that eight EGFDs bear partially hydroxylated asparagine residues; no evidence for hydroxylation of aspartate residues was accrued. The locations of five of the eight identified hydroxylation sites within EGFDs is consistent with the currently accepted criteria for AspH-catalyzed EGFD hydroxylation, that is, N1504 (EGFD17) of latent-transforming growth factor β-binding protein-2 (LTBP2), N877 (EGFD6) of fibulin-2 (FBLN2), N311 (EGFD5) of EGF-containing fibulin-like extracellular matrix protein-1 (EFEMP1; fibulin-3), and N1088 (EGFD16) and N1463 (EGFD25) of human fibrillin-1 (FBN1) appeared to be hydroxylated (Fig. S1). The locations of the remaining three EGFD hydroxylation sites, however, are inconsistent with the currently accepted criteria for AspH-catalyzed EGFD hydroxylation, that is, N281 (EGFD3) of fibulin-1 (FBLN1), N61 (EGFD1) of fibulin-5 (FBLN5), and N5166 (EGFD2) of hemicentin-1 (HMCN1; fibulin-6) appeared to be hydroxylated.

Asparagine residue hydroxylation of four additional FBN1 EGFDs was also supported by analysis of the proteomic mass spectrometry (MS) data, *i.e.*, of N818 in EGFD13, of N1256 in EGFD20, of N2223 in EGFD38, and of N2502 in EGFD43. The locations of these four hydroxylation sites within EGFDs are consistent with the currently accepted criteria for AspH-catalyzed EGFD hydroxylation. However, the MS-MS spectra for these potential hydroxylation sites were of much poorer quality; hence, their assignments should be regarded as provisional. Importantly, these analyses revealed no evidence for hydroxylation of seven EGFD asparagine residues for which (partial) hydroxylation might be anticipated based on the currently accepted criteria for AspH-catalyzed EGFD hydroxylation, that is of FBN1 (*i.e.*, N264, N306, N506, N589, and N1298), FBN3 (*i.e.*, N1256), and FBLN3 (*i.e.*, N192) (Fig. S2*A*).

The latent-transforming growth factor β-binding protein-1 (LTBP1) has been previously identified as containing two hydroxylated asparagine residues (68), whereas our analyses with ovary-derived proteomic data imply that LTBP2 may contain only one hydroxylated asparagine residue (Table 1, entry 1). EGFD aspartate and asparagine residue hydroxylation has been reported for FBLN1 (63), but to our knowledge, not for the structurally related FBLN2 and EFEMP1 (Table 1, entries 2 and 3). Partial aspartate and asparagine hydroxylation has been reported for N1826, D1867, N1949, and N2031 of FBN1 (61), but not for N1088 and N1463 of FBN1. Our analysis provides evidence for high levels of hydroxylation of both N1088 and N1463 of FBN1 (Table 1, entries 4 and 5). For those five EGFD asparagine residues, the extent of their hydroxylation in the reported human ovary proteome was estimated using label-free quantitation analysis (83) (Table 1). The corresponding nonhydroxylated (base) EGFD peptides of N1504 of LTBP2, N311 EFEMP1, and N1088 FBN1 were not detected, suggesting highly efficient asparagine hydroxylation in the human ovary. It should, however, be noted that the stabilities of hydroxylated and nonhydroxylated proteins may differ in cells.

**Table 1:** 

**Figure 3:**
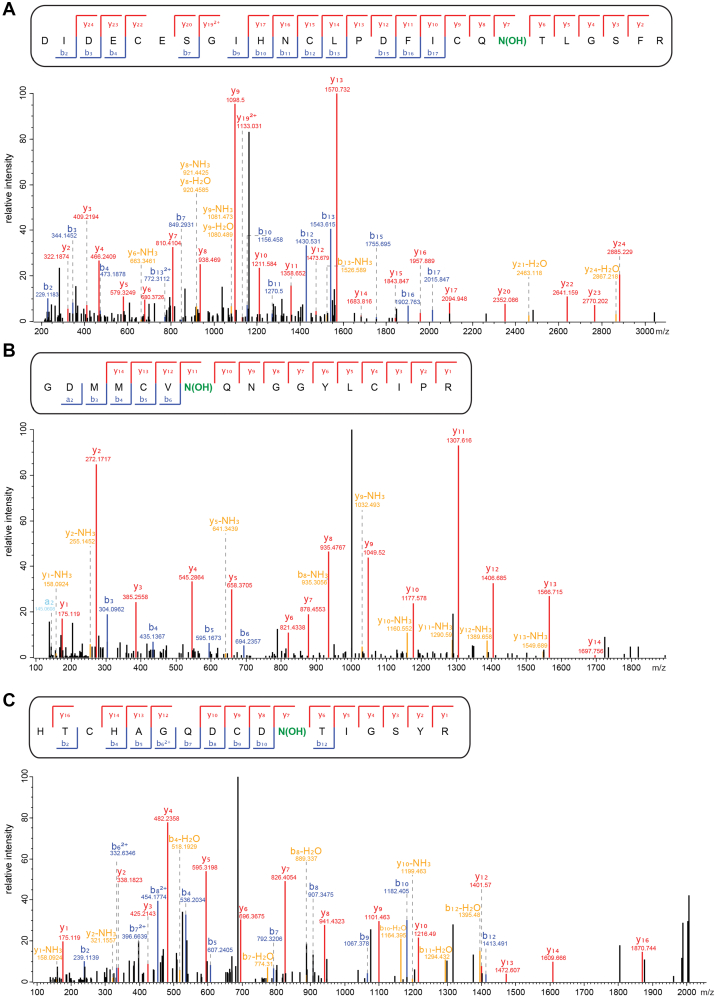
**Analysis of a human ovary-derived proteome** (82) **reveals clear evidence for partial asparagine hydroxylation in EGFDs lacking the C4-X-C5 motif.***A*, MS-MS spectrum for an ovarian FBLN1 EGFD3-derived peptide indicating hydroxylation of N281; the m/z values for y_7_ and higher fragments show a ∼16 Da change. *B*, MS-MS spectrum for an ovarian FBLN5 EGFD1-derived peptide indicating hydroxylation of N61; the m/z values for y_11_ and higher fragments show a ∼16 Da change. *C*, MS-MS spectrum for an ovarian HMCN1 EGFD2-derived peptide indicating hydroxylation of N5166; m/z values for y_7_ and higher fragments, as well as b_12_, show a ∼16 Da change. Assigned potential hydroxylation sites are in *green*. MS spectra of the corresponding nonhydroxylated (base) peptide used to calculate the percentages of EGFD asparagine hydroxylation are shown in Fig. S3. EGFD, epidermal growth factor-like domain; MS, mass spectrometry.

**Figure 4:**
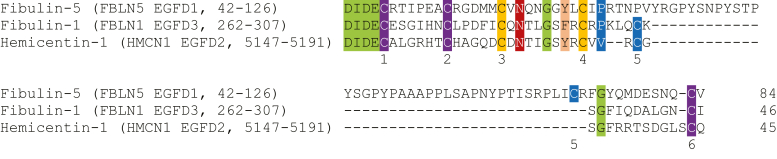
**Sequence alignment of EGFD proteins present in the human ovary identified to bear partially hydroxylated asparagine residues that do not contain a C4-X-C5 motif.** Sequences of FBLN5 EGFD1 (residues 42–126; N61 is partially hydroxylated; UniProt ID: Q9UBX5), FBLN1 EGFD3 (residues 262–307; N281 is partially hydroxylated; UniProt ID: P23142), and HMCN1 EGFD2 (residues 5147–5191; N5166 is partially hydroxylated; UniProt ID: Q96RW7). Partially hydroxylated asparagine residues are in *red*; consensus sequence residues are in *orange* and *salmon*; aligned cysteine residues are in purple, other aligned residues are in *green*; the C5 residue and the 10th residue following the hydroxylation sites are in *blue*; cysteine residues are numbered according to their relative position in the EGFD. EGFD, epidermal growth factor-like domain.

Remarkably, three of the eight ovarian EGFDs found by MS-MS to have partially hydroxylated asparagine residues did not bear the C4-X-C5 motif, *i.e.*, N281 (EGFD3) of fibulin-1 (FBLN1), N61 (EGFD1) of fibulin-5 (FBLN5), and N5166 (EGFD2) of hemicentin-1 (HMCN1; fibulin-6) (Figs. 3 and 4). Human fibulins 1 to 8 are extracellular matrix–associated secreted glycoproteins that bear multiple EGFDs and have diverse functions in extracellular matrix stabilization and remodeling (80, 84, 85), including in relation to wound healing (86). EGFDs in human FBLN1 have been previously reported to be hydroxylated; however, the previous report did not specify which asparagine and/or aspartate residues of the multiple EGFDs are hydroxylated and which are not (63). Our analysis also reveals that five asparagine residues of ovarian EGFDs from FBLN1 (*i.e.*, N235), FBLN2 (*i.e.*, N783, N1044), FBLN3 (*i.e.*, N352), and cartilage oligomeric matrix protein (*i.e.*, N198) are not hydroxylated, even though their sequences resembles those of the hydroxylated EGFDs lacking the C4-X-C5 motif (Fig. S2*B*).

The calculated percentage of EGFD asparagine hydroxylation indicates that the levels of hFBLN1 EGFD3 N281 and hHMCN1 EGFD2 N5166 hydroxylation are approximately similar (*i.e.*, ∼30%; Table 1), while the assigned levels of hFBLN5 EGFD1 N61 hydroxylation are substantially lower (*i.e.*, ∼5%; Table 1, entry 7). The calculated levels of hFBLN1 EGFD3 N281 and hHMCN1 EGFD2 N5166 hydroxylation are similar to that calculated for hFBLN2 EGFD6 N877 hydroxylation (*i.e.*, ∼35%; Table 1, entry 2); note that hFBLN2 EGFD6 bears the C4-X-C5 motif.

In the EGFD3 of human FBLN1, the C4 and C5 residues are five residues apart, while in EGFD2 of human HMCN1, the C4 and C5 residues are three residues apart (Fig. 4). Interestingly, EGFD1 of human FBLN5 bears an RGD integrin-binding motif and an unusual insertion sequence between C4 and C5 (87); thus, the C4-X-C5 motif is substantially altered with 44 residues separating C4 and C5 (Fig. 4). Note that the FBLN5 EGFD1 fold might not be affected by the 44-residue insert and that C4 and C5 of FBLN5 EGFD1 could still be in spatial proximity. Nonetheless, the proteomic MS data clearly show that the C4-X-C5 motif is not an essential requirement for EGFD hydroxylation. Interestingly, it is reported that N974 of EGFD6 of human LTBP1 is partially hydroxylated (68). Sequence analysis of LTBP1 EGFD6 reveals that the C4 and C5 residues are two residues apart (*i.e.*, C4-E-Y-C5) (68), an observation, which, to our knowledge, has not been subsequently explored.

The analysis of a human placenta-derived proteome (82) suggests that the manifested extent of EGFD asparagine hydroxylation may, at least to some extent, be tissue specific (Table 1). While N877 of FBLN2 is ∼35% hydroxylated in ovary-derived cells, it is only ∼20% hydroxylated in placenta-derived cells (Table 1, entry 2). Similarly, N1463 of FBN1 is ∼75% hydroxylated in ovary-derived cells but is only ∼45% hydroxylated in placenta-derived cells (Table 1, entry 5). By contrast, apparently quantitative levels of EFEMP1 N311 hydroxylation were observed in both ovary- and placenta-derived cells (Table 1, entry 3) and FBLN1 N281 hydroxylation levels appear to be similar in both tissues (∼30% and ∼25%, respectively; Table 1, entry 6); note that FBLN1 N281 is part of FBLN1 EGFD3 that does not bear the C4-X-C5 motif.

The apparent differences in the extents of EGFD asparagine hydroxylation may in part reflect context-dependent different expression levels of the genes encoding for AspH or the AspH substrate proteins in specific tissues; for example, the HMCN1 peptide (Table 1, entry 8) was detected in ovary-derived cells, but not in placenta-derived cells. Other factors, including different rates of protein synthesis and degradation (the latter in a potentially hydroxylation-dependent manner), may also complicate the direct comparison of the proteomic data on the extent of hydroxylation, which should thus be interpreted with caution with respect to quantitative correlation with the efficiency of AspH-catalyzed hydroxylation.

### AspH catalyzes the hydroxylation of fibulin EGFD-derived cyclic peptides *in vitro*

To investigate whether AspH catalyzes the observed hydroxylations of human FBLN1 EGFD3, FBLN5 EGFD1, and HMCN1 EGFD2, *in vitro* studies with synthetic thioether-bridged cyclic peptides and purified recombinant human AspH were initiated. Three thioether-bridged cyclic peptides, *i.e.*, hFBLN1-CP_279–297_, hFBLN5-CP_59–77_, and hHMCN1-CP_5164–5182_ (Fig. S4), which mimic the central C3-C4 EGFD disulfide connectivity required for productive AspH catalysis (Fig. 2), were designed based on the corresponding protein sequences and synthesized using solid phase peptide synthesis (SPPS) followed by macrocyclic thioether formation as reported for validated AspH substrates (41, 49).

**Figure 5:**
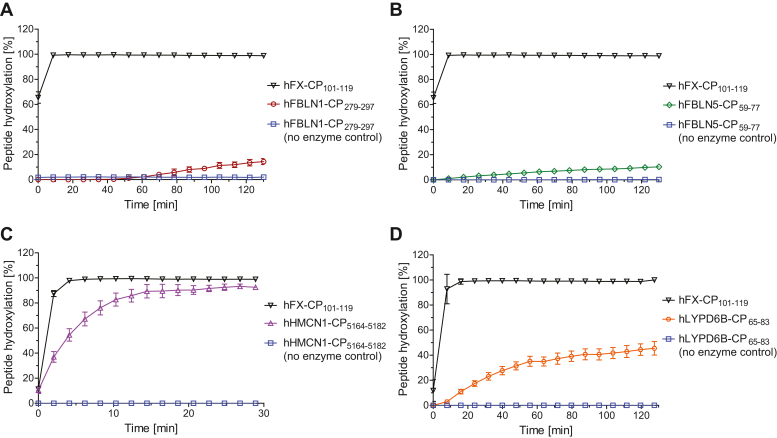
**AspH catalyzes the hydroxylation of cyclic peptides derived from EGFDs which do not bear the C4-X-C5 motif.** Time-course data of the AspH-catalyzed hydroxylation of (*A*) N281 of hFBLN1-CP_279–297_ (*red circles*), (*B*) N61 of hFBLN5-CP_59–77_ (*green diamonds*), (*C*) N5166 of hHMCN1-CP_5164–5182_ (*lavender triangles*), and (*D*) N67 of hLYPD6B-CP_65–83_ (*orange circles*), each compared to hFX-CP_101–119_ (*black inverse triangles*) and a no enzyme control (*blue boxes*); SPE-MS assays were performed in buffer (50 mM Hepes, pH 7.5, rt) as described in the Experimental procedures. Peptide structures are shown in Fig. S4; results are means of three independent runs (n = 3; mean ± SD). AspH, aspartate/asparagine-β-hydroxylase; EGFD, epidermal growth factor-like domain; hFX, human coagulation factor X.

The synthetic cyclic peptides were incubated with recombinant human AspH (His_6_-AspH_315–758_) (41) and peptide hydroxylation was monitored using solid phase extraction coupled to MS (SPE-MS) (49). The results reveal that AspH catalyzes the hydroxylation of the three synthetic cyclic peptides *in vitro*, as evidenced by a +16 Da mass shift compared to a negative (*i.e.*, no enzyme) control. The AspH-catalyzed hydroxylations of hFBLN1-CP_279–297_ and hFBLN5-CP_59–77_ were less efficient than that of the reported synthetic cyclic peptide AspH substrate hFX-CP_101–119_ (41), which mimics the EGFD1 C3–C4 bridged macrocycle of hFX (20, 21) and which was used as a positive control in this study (<20% conversion in 2 h, Fig. 5, *A* and *B*). Remarkably, the AspH-catalyzed hydroxylation of hHMCN1-CP_5164–5182_ (>80% conversion in 10 min, Fig. 5*C*) was substantially more efficient than those of hFBLN1-CP_279–297_ and hFBLN5-CP_59–77_, however, it was still less efficient than that of hFX-CP_101–119_ (>90% conversion in 5 min, Fig. 5*C*).

The results with isolated AspH and synthetic peptides support the proposal based on proteomic analyses that AspH catalyzes the hydroxylation of human FBLN1 EGFD3, FBLN5 EGFD1, and HMCN1 EGFD2 in cells (Fig. 3). The finding that the EGFDs of human FBLN1, FBLN5, and HMCN1 are hydroxylated by AspH invalidates the currently accepted consensus sequence requirements for AspH catalysis, because these substrates lack the previously identified C4-X-C5 consensus motif as C4 and C5 are separated by more than one residue in their EGFDs (Fig. 4). This conclusion raises the question if AspH can potentially catalyze the hydroxylation of substrates other than EGFDs that contain a disulfide-bridged 10 amino acid residue–membered macrocycle.

### AspH catalyzes the hydroxylation of non-EGFD–derived cyclic peptides *in vitro*

Analysis of reported structures of disulfide-rich proteins reveals that the lymphocyte antigen (Ly)-6/plasminogen activator urokinase receptor (PLAUR) domain (LU domain) containing protein 6B (LYPD6B, also known as LYPD7) contains a disulfide-bridged 10 amino acid residue–membered macrocycle on its surface, which apparently fits the revised AspH substrate requirements (Fig. S5) (88, 89). LYPD6B belongs to the LY6/urokinase-type plasminogen activator receptor (uPAR) superfamily, members of which are disulfide-rich and contain a LU domain, but which do not contain EGFDs (89, 90, 91, 92). LYPD6B bears a C-terminal signaling sequence for glycosylphosphatidylinositol-anchoring, indicating that it is processed through the endoplasmic reticulum to be exported to the cell surface (88, 89). It is reported that *LYPD6B* is expressed in the testes, prostate, stomach, lung, and glutamatergic neurons (89, 93) and that LYPD6B modulates specific nicotinic acetylcholine receptors (94); upregulated levels of LYPD6B have been associated with ovarian cancer (95). Given the structure of LYPD6B and the general interest in its biology, proof-of-principle experiments were carried out to investigate whether recombinant AspH can catalyze the *in vitro* hydroxylation of N67 in a LYPD6B-derived cyclic peptide, *i.e.*, hLYPD6B-CP_65–83_ (Fig. S4).

Indeed, AspH-catalyzed hydroxylation of N67 was observed in hLYPD6B-CP_65–83_ by SPE-MS, as supported by comparisons with negative and positive AspH controls (Fig. 5*D*). While the AspH-catalyzed hydroxylation of hLYPD6B-CP_65–83_ was less efficient than that of hFX-CP_101–119_ and hHMCN1-CP_5164–5182_ (∼45% conversion after 2 h), it was more efficient than those of hFBLN1-CP_279–297_ and hFBLN5-CP_59–77_ (Fig. 5). The results support the proposal that AspH can, at least in principle, catalyze the hydroxylation of human substrates other than those with EGFD folds.

Despite being a comparatively efficient AspH substrate *in vitro*, analysis of a human fallopian proteome, in which low levels of LYPD6B were reported (82), did not provide evidence that AspH catalyzes the hydroxylation of LYPD6B N67 in this tissue, despite observation of the corresponding nonhydroxylated base peptide. This discrepancy could be a result of differential expression patterns of AspH and LYPD6B, the low abundance of LYPD6B in this tissue, or it may reflect a preference of AspH for EGFDs determined by factors not solely involving its catalytic domain.

### Determination of kinetic parameters

**Figure 6:**
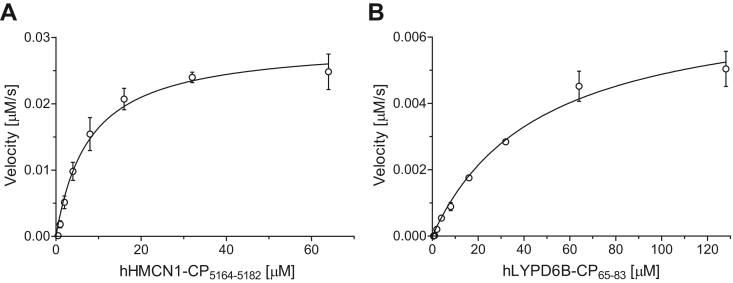
**Determination of steady-state kinetic parameters for the AspH-catalyzed hydroxylations of hHMCN1-CP**_**5164–5182**_**and hLYPD6B-CP**_**65–83**_**.***A*, determination of $v_{max}$ and $K_{m}$ for hHMCN1-CP_5164–5182_; (*B*) determination of $v_{max}$ and $K_{m}$ for hLYPD6B-CP_65–83_. SPE-MS assays were performed as described in the Experimental procedures. Structures of hHMCN1-CP_5164–5182_ and hLYPD6B-CP_65–83_ are shown in Fig. S4, initial hydroxylation rates used to determine kinetic parameters are shown in Figs. S6 and S7; results are means of three independent runs (n = 3; mean ± SD). AspH, aspartate/asparagine-β-hydroxylase.

Kinetic studies were initiated to quantify the efficiency of the cyclic peptides of the new AspH substrates. The determination of AspH kinetic parameters for hFBLN1-CP_279–297_ and hFBLN5-CP_59–77_ using SPE-MS was compromised by their low reactivity with AspH. By contrast, Michaelis constants ($K_{m}$) and maximum velocities ($v_{max}$) of AspH could be accurately determined for hHMCN1-CP_5164–5182_ and hLYPD6B-CP_65–83_ (Fig. 6) and were compared to those reported for hFX-CP_101–119_ (Table 2), which have been previously determined using SPE-MS assays (49).

**Table 2:** Steady-state kinetic parameters of AspH for hHMCN1-CP_5164–5182_, hLYPD6B-CP_65–83_, and hFX-CP_101–119_^a^

Entry	AspH substrate	$v_{max}$ [μM·s^−1^]	$k_{cat}$ [s^−1^]	$K_{m}$ [μM]	$k_{cat}/K_{m}$ [mM^−1^·s^−1^]
1	bhFX-CP_101–119_	0.019 ± 0.002	0.20 ± 0.03	1.3 ± 0.3	160 ± 40
2	hHMCN1-CP_5164–5182_	0.029 ± 0.002	0.30 ± 0.05	7.9 ± 1.0	38 ± 8
3	hLYPD6B-CP_65–83_	0.007 ± 0.001	0.07 ± 0.02	47 ± 6	1.5 ± 0.5

a Determined using 0.1 μM His_6_-AspH_315–758_, 100 μM LAA, 20 μM 2OG, and 20 μM Fe(II) in buffer (50 mM Hepes, pH 7.5, 20 °C) as described in the Experimental procedures. Results are means of three independent runs (n = 3; mean ± SD).

b The kinetic parameters of AspH for hFX-CP_101–119_ have been previously determined under the same conditions using SPE-MS (49).

Turnover numbers ($k_{cat}$) of AspH for hHMCN1-CP_5164–5182_ and hLYPD6B-CP_65–83_ were calculated from the $v_{max}$ values, assuming that the fraction of purified AspH that is active is 95.1 ± 14.3%, as determined by an active site titration analysis as reported (49). Comparison of the $k_{cat}$ values reveals that, within error, turnover of saturating amounts of hLYPD6B-CP_65–83_ is approximately threefold and fourfold slower with respect to hFX-CP_101–119_ and hHMCN1-CP_5164–5182_, respectively (Table 2). In general, differences in the $K_{m}$ values for hHMCN1-CP_5164–5182_, hLYPD6B-CP_65–83_, and hFX-CP_101–119_ appear to be more pronounced than their respective $k_{cat}$ values. The $K_{m}$ values of AspH for hHMCN1-CP_5164–5182_ and hLYPD6B-CP_65–83_ are ∼6-fold and ∼36-fold, respectively, higher than that reported for hFX-CP_101–119_ (Table 2), which indicates a lower affinity of AspH for hHMCN1-CP_5164–5182_ and, in particular, hLYPD6B-CP_65–83_ compared to hFX-CP_101–119_.

The $k_{cat}/K_{m}$ value reported for hFX-CP_101–119_ with AspH (49) is about fourfold higher than that determined for hHMCN1-CP_5164–5182_ and about two orders of magnitude higher than that determined for hLYPD6B-CP_65–83_ (Table 2). The $k_{cat}/K_{m}$ values thus appear to reflect the results of the proteomic analysis, which showed that D103 in the EGFD1 of hFX is apparently fully hydroxylated in humans, while N5166 in the EGFD2 of hemicentin-1 (HMCN1) is only ∼30% hydroxylated (Table 1). Although, as noted previously, multiple other factors may be involved in regulating substrate turnover; this observation also correlates with the apparent lack of evidence for N67 hydroxylation in LYPD6B in human fallopian tube cells.

## Discussion

**Figure 7:**
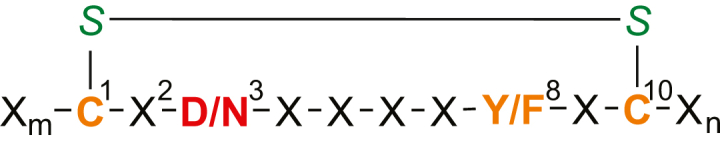
**Minimal substrate requirements for productive AspH catalysis.** The AspH hydroxylation site is in *red*, consensus sequence residues are in *orange*, and cystine sulfurs are in *green*. ‘X’ represents other proteinogenic amino acids; note, the presence of the preferred substrate requirements in EGFDs does not always correlate with hydroxylation in cells, as observed by MS analyses. AspH, aspartate/asparagine-β-hydroxylase; MS, mass spectrometry.

Our combined *in vitro* studies and proteomic analyses demonstrate that the substrate requirements for, at least efficient, productive AspH catalysis should be revised to comprise a disulfide-bridged 10 residue–membered macrocycle (for EGFDs: C3–C4) in which the third residue is either D or N (the site of AspH-catalyzed hydroxylation) and the eighth residue is either F or Y (Fig. 7), a residue shown by crystallographic analyses to directly interact with the TPR domain of AspH (Fig. 1*E*) (41). An EGFD C4-X-C5 motif is not required for EGFD asparagine residue hydroxylation in cells and *in vitro* (catalyzed by recombinant human AspH) (Figure 3, Figure 4, Figure 5 and Table 1, Table 2, Table 3). The revised EGFD substrate requirements for AspH catalysis were validated by the identification of three hydroxylated asparagine residues in EGFDs of fibulins, *i.e.*, N281 in fibulin-1, N61 in fibulin-5, and N5166 in hemicentin-1 (Fig. 3), in proteomic MS data from human ovary tissue. These residues would not have been predicted to be hydroxylated based on the previously accepted EGFD substrate requirements for AspH catalysis. It should be noted, however, that the presence of the revised substrate requirements for AspH catalysis does not necessarily result in the observation of EGFD hydroxylation. Thus, other structural features of EGFDs, which are presently poorly understood, must govern the degree of hydroxylation of particular EGFDs *in vivo*, the extent of which varies considerably from an apparent lack to apparently quantitative levels of hydroxylation. This observation might in part reflect the low sequence similarity in EGFDs (1).

**Table 3:** Summary of hydroxylated asparagine residues in EGFDs lacking the C4-X-C5 motif

Entry	Protein	Site of hydroxylation	Domain	Apparent hydroxylation in ovary-/placenta-derived cells	Hydroxylation of EGFD/LU domain-derived synthetic peptides by recombinant human AspH
1	FBLN1	N281	EGFD3	∼30%/∼25%	<20% after 2 h
2	FBLN5	N61	EGFD1	∼5%/∼0%	<20% after 2 h
3	HMCN1	N5166	EGFD2	∼30%/not detected	>80% in 10 min
4	LYPD6B	N67	LU1	∼0%^a^	∼45% after 2 h

a Low levels of (nonhydroxylated) LYPD6B were detected in fallopian tube-derived cells, but not in ovary- and placenta-derived cells.

The results with isolated recombinant human AspH reveal that it catalyzes the hydroxylation of EGFDs with a C3–C4 disulfide-bridged macrocycle, but not of isomeric disulfide-bridged macrocycles, including the C2–C4 disulfide-bridged macrocycle, which is part of the ‘canonical’ (*i.e.*, C1–C3, C2–C4, and C5–C6) EGFD disulfide pattern that has been observed in most (4, 5, 6, 7, 8), but not all (71, 72, 73, 74), EGFD structures (Fig. 2). The biological significance of this observation remains unclear, partly because of the current lack of animal models to investigate the phenotype of AspH-catalyzed EGFD hydroxylation (28). We have proposed an as yet unvalidated role for AspH in the regulation of EGFD folding in the endoplasmic reticulum (41), which could extend to non-EGFD–containing disulfide-rich proteins bearing the revised sequence for AspH-catalyzed hydroxylation (Fig. 7). It should also be noted that a soluble N-terminally truncated AspH construct, which only contains the catalytic oxygenase and TPR domains, was used in our studies. In principle, it is possible that additional domains in AspH, including the Ca(II)-binding EF-hand domain, may regulate the selectivity of AspH catalysis including by altering the EGFD disulfide requirements for productive AspH catalysis.

The availability of large-scale proteomic MS data in public repositories such as Pride (96) was vital to our work. Assignments employing multinotch software such as MetaMorpheus (77) enable searches of the entire human proteome of a cell line or tissue accommodating dozens of potential PTMs, which can now be accomplished using desktop computers. Incorrect assignments can occur, for example due to an incomplete series of b or y ions in the data, by the presence of unassigned modifications, or by the presence of ions from coeluting peptides with precursor masses within 2 Da of the mass of the major component. For this reason, hydroxylated peptide structures obtained from search engines should be considered as potential PTMs, even after inspection of the MS data by experts. We thus suggest that validating PTM assignments by synthesis of standards and/or by studies with isolated enzymes and potential substrates is desirable to reduce misidentifications, in particular for challenging PTMs such as hydroxylation. We also note that tissue culture conditions likely often do not reflect the *in vivo* context, in particular with respect to oxygen availability, which can impact on the extent of protein (or other biomolecule) hydroxylation; this may be particularly so for enzymes such as AspH with apparently high $K_{m}$ values for oxygen (49). For this reason, proteomic analyses of samples from human tissues rather than of cultured cells are preferable where possible.

Our work has identified EGFDs in human fibulins as substrates of AspH-catalyzed asparagine hydroxylation in the ovary, an observation which is of interest considering the importance of fibulins in extracellular matrix biochemistry, including their proposed roles in cancer biology (80, 84). Fibulins might be relevant AspH downstream targets transmitting pathophysiologic-relevant effects of AspH upregulation in some cancer cells. Studies with isolated recombinant AspH and synthetic substrates revealed the potential of AspH to catalyze the observed EGFD hydroxylations in fibulins. Previous work has shown that the functionally characterized human asparagine residue hydroxylase factor inhibiting HIF-α (FIH) does not accept EGFDs as substrates *in vitro* (97). However, other enzymes, including the structurally and functionally uncharacterized aspartate β-hydroxylase domain–containing proteins 1 or 2 (AspHD1/2), which likely fold in a similar manner as the AspH catalytic domain (43), could potentially catalyze the hydroxylation of fibulin EGFDs (and other substrates), possibly in a more efficient manner than AspH—a possibility that requires further *in vitro* validation with respect to all reported AspH substrates.

Apart from fibulin EGFDs, the EGFDs of human fibrillin-1 (FBN1) are the other prominent identified AspH substrates in the ovary, an observation which may be of relevance from a disease perspective. Partial asparagine hydroxylation has been reported for FBN1 N1826 (61); the FBN1 N1826S substitution has been reported in a 16-year-old patient diagnosed with Marfan syndrome, which is associated with aortic dilatation and mitral valve prolapse (98). We observed partial hydroxylation of FBN1 N1088; the N1088I substitution in a <1-year-old patient diagnosed with a severe form of Marfan syndrome has been reported to have major effects on the cardiovascular system (99). It is unclear whether the Marfan phenotype associated with the FBN1 N1088I and N1826S variants is a direct consequence of the absence of asparagine hydroxylation. Alternatively, the phenotype may reflect changes in the EGFD fold or be due to the modulated ability of the EGFD to coordinate Ca(II) (100). Several FBN1 EGFDs bind Ca(II) in a manner that involves direct coordination of the (hydroxy-)aspartate/asparagine side chain carboxylate/carboxamide to Ca(II) (101). Ca(II) binding is reported to rigidify the EGFD quaternary structure (102, 103, 104, 105) and stabilize EGFDs against proteolytic degradation (106, 107). However, it is poorly understood how Ca(II) binding in EGFDs affects AspH catalysis (41, 108), though it may do so, for example, by regulating the availability of the noncanonical disulfide pattern that we have found is an AspH substrate. Nonetheless, the clinical observations indicate a potential function for FBN1 EGFD asparagine hydroxylation in signaling and extracellular matrix stabilization (109).

While the analysis of human proteomes for potential AspH substrates, which do not contain EGFDs, has so far not resulted in the identification of such substrates, the results with isolated proteins show that cellular substrates other than EGFDs that fulfill our revised substrate requirements for AspH catalysis might, at least in principle, exist. In particular, N67 of human LYPD6B, which is a disulfide-rich protein that does not contain EGFDs, was hydroxylated by purified recombinant human AspH (Fig. 5*D*). However, the analysis of a human fallopian proteome revealed no evidence of LYPD6B N67 hydroxylation in this tissue; further work is required to investigate whether LYPD6B N67 is hydroxylated in other tissues. Nonetheless, the results clearly highlight the potential of AspH to catalyze the hydroxylation of substrates other than EGFDs, which is of interest considering that catalytically active AspH has been detected on the surface of certain cancer cells (110), where it can, in principle, hydroxylate proteins to which it is not exposed in healthy cells.

The proposal that AspH catalyzes the hydroxylation of asparagine and aspartate residues present in domains other than EGFDs is precedented by research on other human 2OG oxygenases that accept multiple different substrates, for example, the asparagine residue hydroxylase FIH. FIH was originally identified to suppress HIF-mediated transcription by catalyzing the C3 hydroxylation of an asparagine residue in the C-terminal transactivation domain of HIF-α isoforms (N803 in HIF-1α) (111). Later, it was shown that FIH can also catalyze the hydroxylation of asparagine and other residues in ankyrin repeats (112, 113, 114, 115, 116). A role for FIH-catalyzed HIF-α hydroxylation in the hypoxic response has been identified, *i.e.*, it regulates the expression of HIF target genes by hindering the interaction between HIF and the CBP/p300 histone acetyl transferase (117). However, a physiologically relevant role for FIH-catalyzed ankyrin hydroxylation has not been identified, as is the case for AspH-catalyzed EGFD hydroxylation.

The extents of AspH and FIH catalyzed hydroxylation of their different EGFD and ankyrin substrates, respectively, varies substantially (20, 21, 113, 114, 115, 118). Ongoing work also indicates that the extent of AspH-catalyzed EGFD hydroxylation may vary in different tissues (Table 1). FIH-catalyzed ankyrin hydroxylation can stabilize the ankyrin fold (119), but given the varying levels of ankyrin and EGFD hydroxylation observed, this would seem unlikely to be a general role. One possible function of hydroxylation relates to competition between directly signaling and nondirectly signaling substrates for the active sites of FIH and AspH that helps to robust signaling. Thus, for FIH, competition between HIF-α (direct signaling) and ankyrin/other substrates (nondirect signaling, at least in the context of the hypoxic response) (120), may occur, though experimental validation of the physiological relevance for this proposal is currently lacking. It is also possible that the roles of 2OG-dependent protein hydroxylases with multiple substrates relate to their use of Fe(II) as a cofactor and 2OG, CO_2_, and succinate as cosubstrates/coproducts, *i.e.*, they may be involved in the regulation of small-molecule metabolism/redox biochemistry in cells. Additionally, it should be noted that unidentified roles for AspH-catalyzed hydroxylation in signaling may manifest in developmental or environmental contexts, which have not yet been examined.

Despite apparent complexities in the roles of 2OG oxygenases in post-translational protein hydroxylation, targeting them is possible from a medicinal chemistry perspective. Indeed, HIF-α prolyl hydroxylase inhibitors are used for the treatment of anemia (121). By informing on the cellular substrates of AspH, we hope that the results presented here will help enable the development of potent and selective small-molecule inhibitors of AspH, which is of interest from a cancer treatment perspective (54, 55, 122, 123, 124, 125, 126, 127).

## Experimental procedures

### General

Assay buffers and cosubstrate/cofactor stock solutions (L-ascorbic acid, LAA; 2OG; ammonium iron(II) sulfate hexahydrate, FAS, (NH_4_)_2_Fe(SO_4_)_2_·6H_2_O) were freshly prepared from commercially sourced solids using MQ-grade water.

### Analysis of human proteomes

LC-MS-MS raw data was obtained from the ProteomeXchange using identifier PXD010154 (82). The human FASTA database was obtained from UniProt as of December 25, 2021, and contained 20,387 protein sequences. The FASTA formatted contaminant database was downloaded from thegpm.org (January 01, 2012) and contained 115 sequences. A global PTM discovery (GPTM-D) search was performed using MetaMorpheus software (revision 0.0.320) available at https://github.com/smith-chem-wisc/MetaMorpheus (77). The parameters used for MetaMorpheus searches were the same as previously described (78); PTMs other than hydroxylation, such as glycosylations, were included in the proteomic searches. The results were filtered to a global false discovery rate of 1% and a notch false discovery rate of 1%. Data files were processed on a Dell Inc. XPS 15 7590 computer, using Windows 10 PRO version 20H2 with a 64-Bit processor with four cores operating at 2.6 GHz and 32 GB installed random access memory. The three tasks combined required ∼18 h of computer time for the 36 RAW files.

The apparent percentages of EGFD asparagine hydroxylations (site occupancy) were estimated using label-free quantitation analysis only for those peptides for which the non-hydroxylated base peptides could also be detected (83).

### AspH production and purification

An N-terminally truncated construct of WT N-terminally His_6_-tagged human AspH, comprising the catalytic oxygenase domain and the TPR domain (His_6_-AspH_315–758_), was produced and purified as reported (41, 49).

### Peptide synthesis

The synthesis and purification of hFX-CP_101–119_ has been described (49); the other four thioether-bridged cyclic peptides used in this study, *i.e.*, hFBLN1-CP_279–297_, hFBLN5-CP_59–77_, hHMCN1-CP_5164–5182_, and hLYPD6B-CP_65–83_ (Fig. S4), were synthesized as C-terminal amides in a similar manner. In brief, linear peptides were synthesized from the C to the N terminus on Rink Amide MBHA resin (AGTC Bioproducts Ltd; loading: 0.6–0.8 mmol/g) by microwave-assisted SPPS using an automated peptide synthesizer (Liberty Blue, CEM Microwave Technology Ltd). After coupling of the final N-terminal amino acid, its Fmoc protecting group was cleaved and the resultant free N-terminal amine capped using *N*-chloroacetoxysuccinimide. Peptides were then cleaved from the resin and simultaneously deprotected using a mixture of TFA, triisopropylsilane, 1,3-dimethoxybenzene, and water (92.5/2.5/2.5/2.5%_v/v_, respectively). Solids were separated, and the linear peptides were precipitated by diethyl ether treatment. The linear peptides were lyophilized, dissolved in a mixture of water, acetonitrile, and triethylamine, and cyclized in a microwave reactor (Biotage Initiator) at 80 °C. The crude reaction mixtures were filtered and purified using a semipreparative HPLC machine (Shimadzu UK Ltd) equipped with a reverse-phase column (Gemini 00G-4454-U0-AX; phase: NX-C18). A linear gradient (2–40%_v/v_ over 40 min) of acetonitrile in water (each containing 0.1%_v/v_ TFA) was used as the eluent. Fractions were analyzed by SPE-MS and those containing the purified cyclic peptide were combined and lyophilized. Sequences, mass spectra, and HPLC retention times for the peptides synthesized are shown in Fig. S1. Note that peptide synthesis is assumed to proceed without substantial loss of stereochemistry, as evidenced by crystallographic analysis of a peptide prepared by this procedure in complex with AspH (41).

The four disulfide isomers of the reported AspH substrate hFX-EGFD1_86–124_-4Ser **3** (41, 49) used in this study (Fig. 2), *i.e.*, **1**, **2**, **4**, and **5**, were synthesized by SPPS and purified by GL Biochem Ltd; the peptides were prepared with C-terminal amides.

### AspH MS turnover assays

AspH assays using the hFX-EGFD1_86–124_-4Ser isomers **1** to **5** as substrates were performed in independent triplicates, using 0.15 μM His_6_-AspH_315–758_, 5.0 μM of a pure hFX-EGFD1_86–124_-4Ser disulfide isomer, 100 μM LAA, 20 μM 2OG, and 20 μM FAS in buffer (50 mM Tris, pH 7.5, 50 mM NaCl, 20 °C). AspH assays using the thioether-bridged cyclic peptides hFBLN1-CP_279–297_, hFBLN5-CP_59–77_, hHMCN1-CP_5164–5182_, or hLYPD6B-CP_65–83_ as substrates were performed in independent triplicates using 0.1 μM His_6_-AspH_315–758_, 2.0 μM substrate, 100 μM LAA, 10 μM 2OG, and 10 μM FAS in buffer (50 mM HEPES, 20 °C). Reactions with the thioether-bridged cyclic peptide substrates were performed in parallel with negative (*i.e.*, no enzyme) and positive (*i.e.*, using the reported synthetic cyclic peptide hFX-CP_101–119_ (41), which mimics the EGFD1 C3–C4 bridged macrocycle of hFX (20, 21)) controls.

Reaction progress was monitored using SPE-MS employing a RapidFire RF 365 high-throughput sampling robot (Agilent) attached to an iFunnel Agilent 6550 accurate mass quadrupole time-of-flight mass spectrometer operated in the positive-ionization mode with the following parameters: capillary voltage (4000 V), nozzle voltage (1000 V), fragmentor voltage (365 V), gas temperature (280 °C), gas flow (13 L/min), sheath gas temperature (350 °C), sheath gas flow (12 L/min). Assay samples were aspirated under vacuum, loaded onto a C4 SPE cartridge, and peptides eluted into the mass spectrometer as described (43, 49, 76, 97). For data analysis, the m/z +2 charge states of the thioether-bridged cyclic peptides or the m/z +4 charge states of the hFX-EGFD1_86–124_-4Ser peptides were used to extract ion chromatogram data; peak areas were integrated using RapidFire Integrator software (Agilent). Data were exported into Microsoft Excel and used to calculate the % conversion of the hydroxylation reaction using the equation: % conversion = 100 × (integral product peptide)/(integral substrate peptide + integral product peptide).

### Determination of kinetic parameters

Maximum velocities ($v_{max}$) and Michaelis constants ($K_{m}$) of AspH for hHMCN1-CP_5164–5182_ and hLYPD6B-CP_65–83_ were determined in independent triplicate analyses by directly monitoring hydroxylation using SPE-MS, as reported for related thioether-bridged cyclic peptides (49). In brief, His_6_-AspH_315–758_ was added (0.1 μM final concentration) to a mixture of 100 μM LAA, 20 μM FAS, 20 μM 2OG, and substrate in buffer (50 mM Hepes, pH 7.5, 20 °C); final substrate concentrations are given in Figs. S6 and S7. Reactions were monitored using SPE-MS with the same configuration as described previously. Data were analyzed as described previously and the slopes of the initial reaction rates (Figs. S6 and S7) fitted to a Michaelis–Menten plot using nonlinear regression (GraphPad Prism 5). The total concentration of active AspH has been determined previously by an active site titration (49) and was used to calculate turnover numbers ($k_{cat}$).

## Data availability

All relevant data are located within the article and supporting information. Proteomic data have been obtained from ProteomeXchange using identifier PXD010154 (82) and protein sequences from UniProt.

## Supporting information

This article contains supporting information (10, 29, 41, 46, 49, 56, 57, 58, 82, 83, 88).

## Conflict of interest

The authors declare that they have no conflicts of interest with the contents of this article.
