# Supplementary material for: Combined proteomic and biochemical analyses redefine the consensus sequence requirement for epidermal growth factor-like domain hydroxylation
Source: J Biol Chem. 2022 Jun 11;298(8):102129. doi: 10.1016/j.jbc.2022.102129 (PMC9293771; doi:10.1016/j.jbc.2022.102129)
Supplement: Supplemental Figures S1–S7 [file mmc1.pdf]

Supporting Information

Combined proteomic and biochemical analyses redefine the consensus sequence requirement for epidermal growth factor-like domain hydroxylation

Lennart Brewitz[1,*], Bruce C. Onisko[2,*], and Christopher J. Schofield[1,*]

[1]Chemistry Research Laboratory, Department of Chemistry and the Ineos Oxford Institute for Antimicrobial Research, University of Oxford, 12 Mansfield Road, OX1 3TA, Oxford, United Kingdom.

[2]OniPro LLC, 330 Berkeley Park Blvd., Kensington, CA 94707, United States of America.

*lennart.brewitz@chem.ox.ac.uk, bconisko@gmail.com, christopher.schofield@chem.ox.ac.uk

Supporting Figure S1. AspH-catalyzed asparagine-residue hydroxylation in EGFDs of a reported human ovary-derived proteome (continues on the following three pages). Analysis of a human ovary-derived proteome (1) reveals clear evidence for AspH-catalyzed asparagine hydroxylation in EGFDs, as anticipated based on the reported consensus EGFD requirements for AspH catalysis, *i.e.* C_3-X-D/N-X-X-X-X-F/Y-X-C_4-X-C_5 (2-7). Partial hydroxylation of N1504 (EGFD17) of latent-transforming growth factor beta-binding protein-2 (LTBP2), of N877 (EGFD6) of fibulin-2 (FBLN2), of N311 (EGFD5) of EGF-containing fibulin-like extracellular matrix protein-1 (EFEMP1; fibulin-3), and of N1088 (EGFD16) and N1463 (EGFD25) of human fibrillin-1 (FBN1) was observed. Hydroxylation sites are in green; y ions are in red, b ions are in blue, fragmented ions are in orange.

I. The observed ion fragmentation pattern of a peptide assigned to LTBP2 EGFD17 indicates AspH-catalyzed hydroxylation of N1504. The observed m/z values for b_3 and higher fragments reveal a shift in the m/z of N1504 which corresponds to hydroxylation. The spectrum of the non-hydroxylated (base) peptide of LTBP2 EGFD17 was not detected, in accord with an apparently quantitative (>90%) level of hydroxylation.

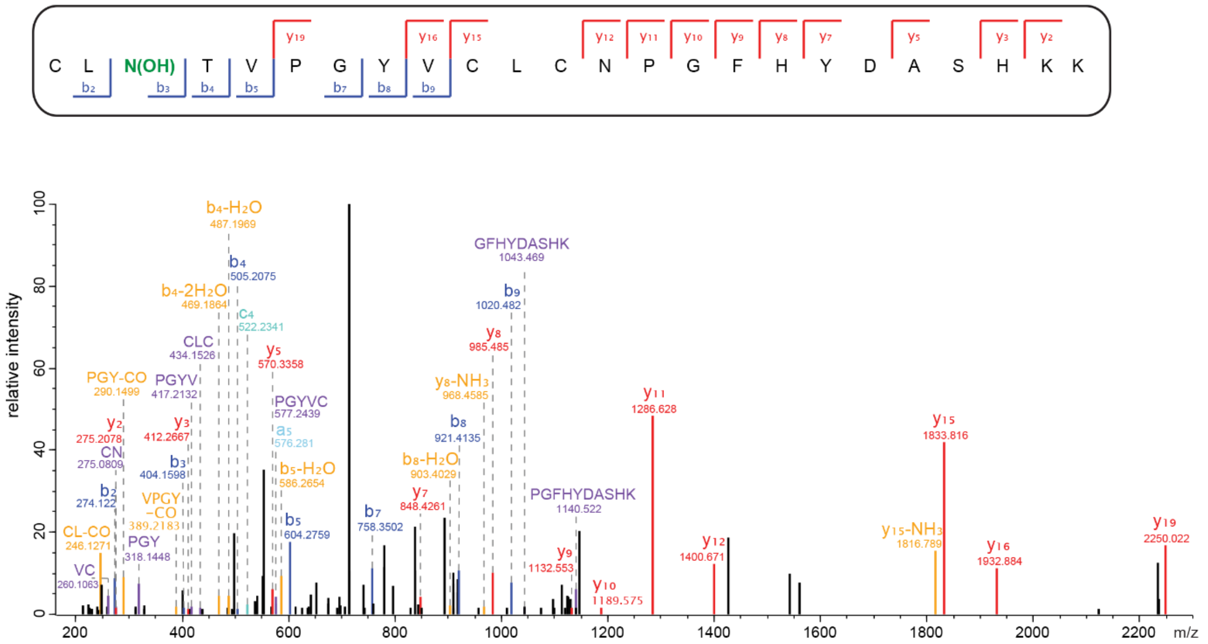

II. The observed ion fragmentation pattern of a peptide assigned to FBLN2 EGFD6 indicates AspH-catalyzed partial hydroxylation of N877. The m/z values for y_6 and smaller fragments of both: (**a**) the non-hydroxylated (base) and (**b**) the hydroxylated peptides of the FBLN2 EGFD6 are identical (*i.e.* the m/z for y_6 is 692.3726), while the observed m/z values for y_7 and higher fragments differ by ~16 for (**a**) the non-hydroxylated (base) and (**b**) the hydroxylated peptides of FBLN2 EGFD6 (*i.e.* m/z values for y_7 are 806.4155 and 822.4104, respectively). Similarly, the m/z values for b_8 and smaller fragments of both (**a**) the non-hydroxylated (base) and (**b**) the hydroxylated peptides of the FBLN2 EGFD6 are identical, while the observed m/z value for b_9 differs by ~16 for (**a**) the non-hydroxylated (base) and (**b**) the hydroxylated peptides of FBLN2 EGFD6 (*i.e.* m/z values of 1042.383 and 1058.378, respectively). Using label free quantitation (LFQ) analysis (8), the percentage site occupancy of hydroxy-asparagine was calculated to be ~36%.

(a)

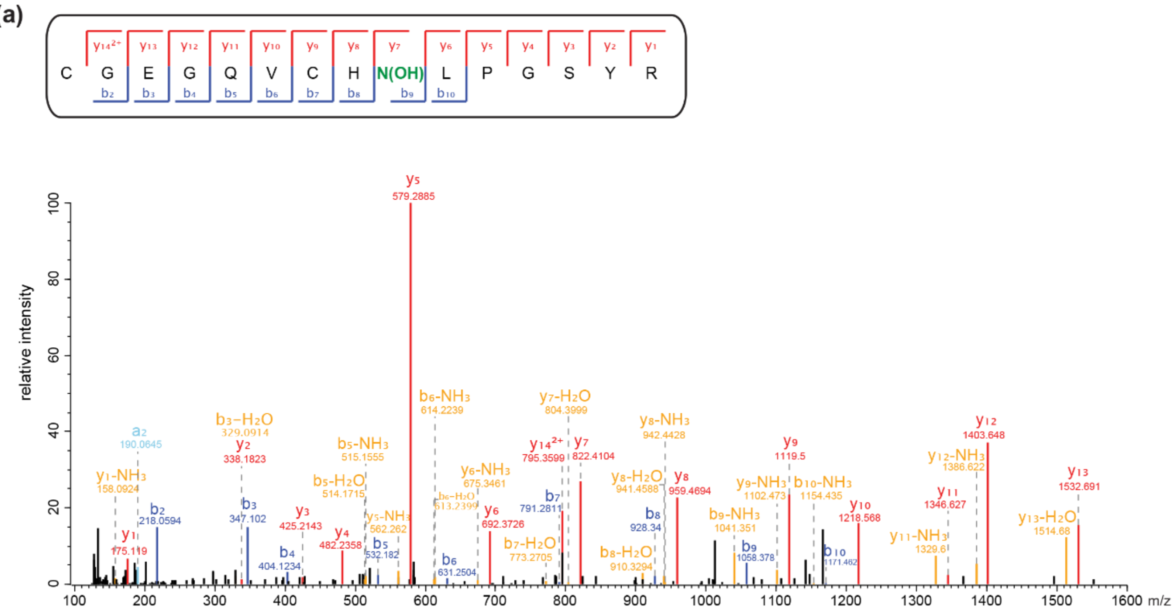

(b)

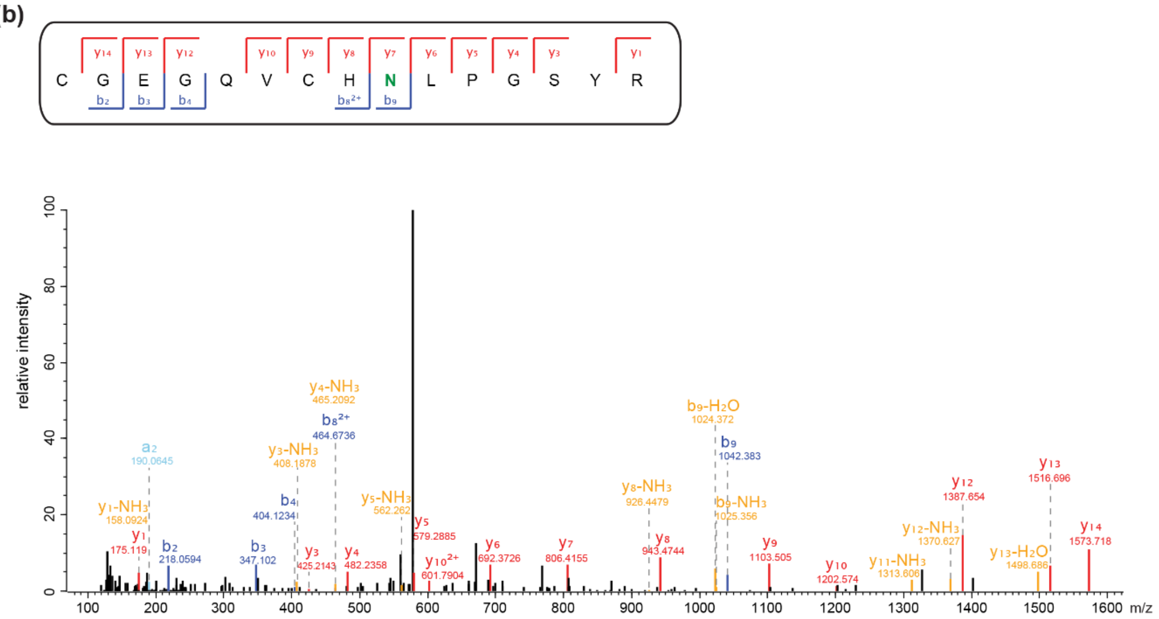

III. The observed ion fragmentation pattern of a peptide assigned to EFEMP1 EGFD5 indicates AspH-catalyzed hydroxylation of N311. The observed m/z values for y_5 and higher fragments reveal a shift in the m/z of N311 which corresponds to hydroxylation. The spectrum of the non-hydroxylated (base) peptide of EFEMP1 EGFD5 was not detected, in accord with an apparently quantitative (>90%) level of hydroxylation.

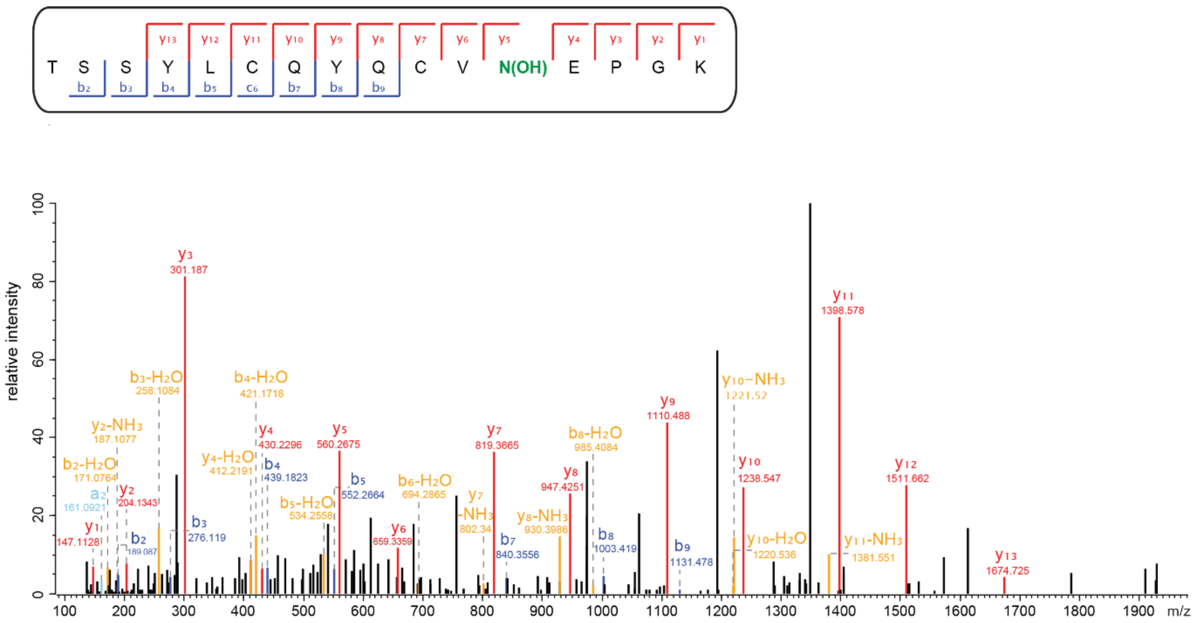

IV. The observed ion fragmentation pattern of a peptide assigned to FBN1 EGFD16 indicates AspH-catalyzed hydroxylation of N1088. The observed m/z values for y_9 and higher fragments, as well as for b_6, reveal a shift in the m/z of N1088 which corresponds to hydroxylation. The spectrum of the non-hydroxylated (base) peptide of FBN1 EGFD16 was not detected, in accord with an apparently quantitative (>90%) level of hydroxylation.

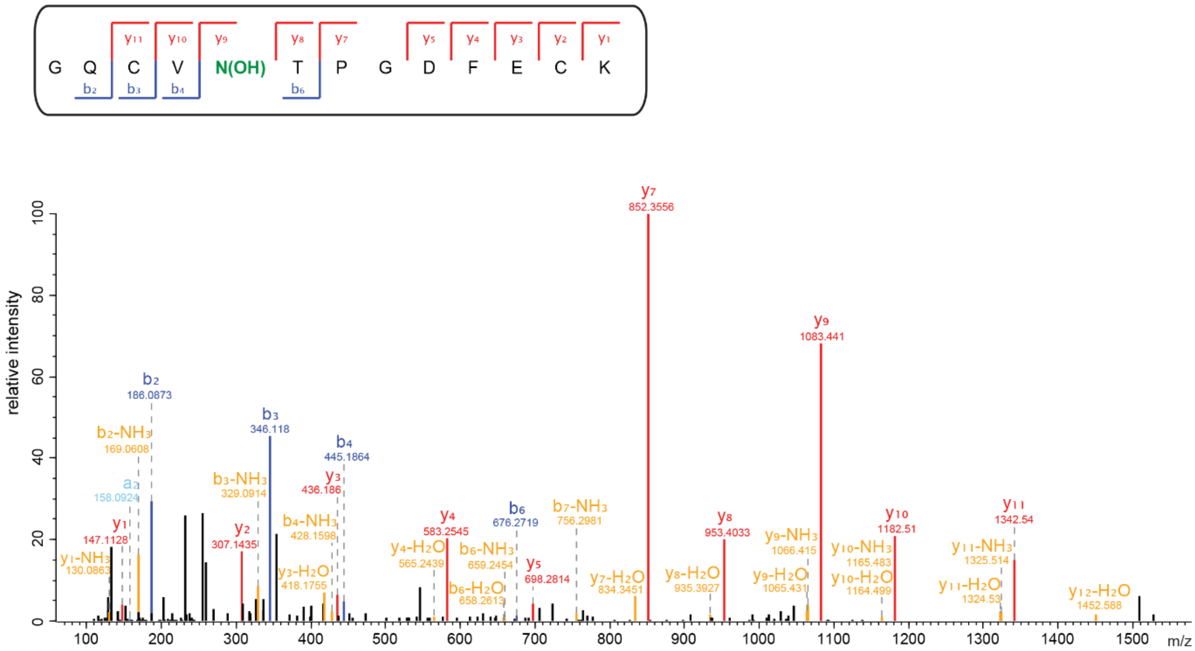

V. The observed ion fragmentation pattern of a peptide assigned to FBN1 EGFD25 indicates AspH-catalyzed partial hydroxylation of N1463. The m/z values for y_6 and smaller fragments of both: (**a**) the non-hydroxylated (base) and (**b**) the hydroxylated peptides of the FBN1 EGFD25 are identical (*i.e.* the m/z for y_6 is 702.4297), while the observed m/z values for y_7 and higher fragments differ by ~16 for (**a**) the non-hydroxylated (base) and (**b**) the hydroxylated peptides of FBN1 EGFD25 (*i.e.* m/z values for y_7 are 816.4726 and 832.4676, respectively). Using label free quantitation (LFQ) analysis (8), the percentage site occupancy of hydroxy-asparagine was calculated to be ~75%.

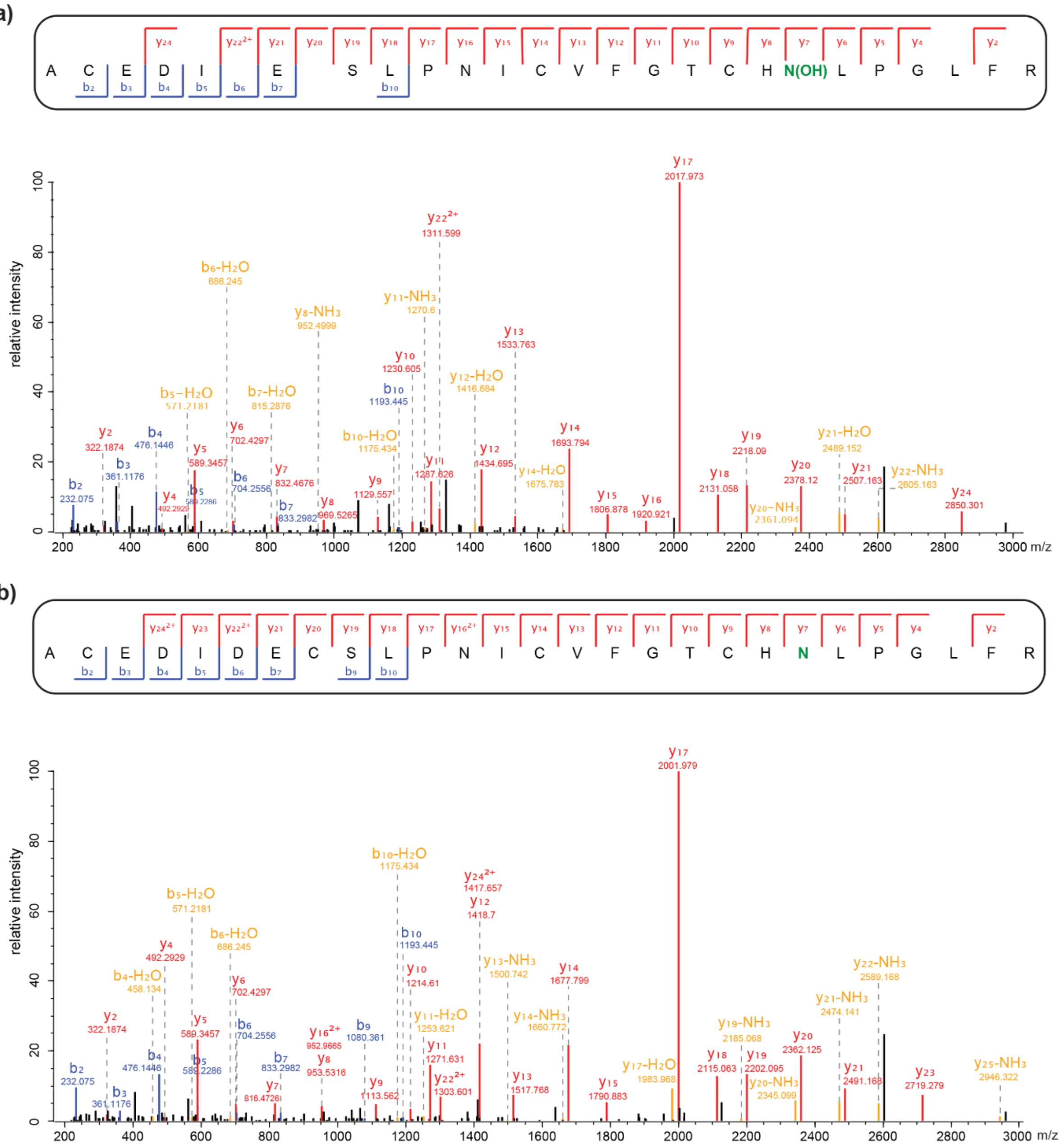

Supporting Figure S2. Sequence alignment of human ovarian EGFDs identified to not bear hydroxylated asparagine residues. (**a**) Sequences of fibrillin-1 EGFD4 (N264 is not hydroxylated; UniProt ID: P35555), fibrillin-1 EGFD5 (N306 is not hydroxylated; UniProt ID: P35555), fibrillin-1 EGFD7 (N506 is not hydroxylated; UniProt ID: P35555), fibrillin-1 EGFD9 (N589 is not hydroxylated; UniProt ID: P35555), fibrillin-1 EGFD21 (N1298 is not hydroxylated; UniProt ID: P35555), fibrillin-3 EGFD18 (N1256 is not hydroxylated; UniProt ID: Q75N90), and fibulin-3 (EGF-containing fibulin-like extracellular matrix protein 1) EGFD2 (N192 is not hydroxylated; UniProt ID: Q12805). Hydroxylation for these seven asparagine residues could be anticipated based on the currently accepted criteria for AspH-catalyzed EGFD hydroxylation (2-7). (**b**) Sequences of fibulin-1 EGFD2 (N235 is not hydroxylated; UniProt ID: P23142), fibulin-2 EGFD4 (N783 is not hydroxylated; UniProt ID: P98095), fibulin-2 EGFD10 (N1044 is not hydroxylated; UniProt ID: P98095), fibulin-3 (EGF-containing fibulin-like extracellular matrix protein 1) EGFD6 (N352 is not hydroxylated; UniProt ID: Q12805), and cartilage oligomeric matrix protein EGFD3 (N198 is not hydroxylated; UniProt ID: P49747). Hydroxylation for these five asparagine residues would not have been anticipated based on the currently accepted criteria for AspH-catalyzed EGFD hydroxylation (2-7), but could be anticipated based on our revised criteria for AspH-catalyzed EGFD hydroxylation (Figure 7).

Asparagine residues anticipated to be hydroxylated are in red; consensus sequence residues are in orange and salmon; aligned cysteine residues are in purple, other aligned residues are in green; the aligned C5 residues are in blue; cysteine residues are numbered according to their relative position in the EGFD.

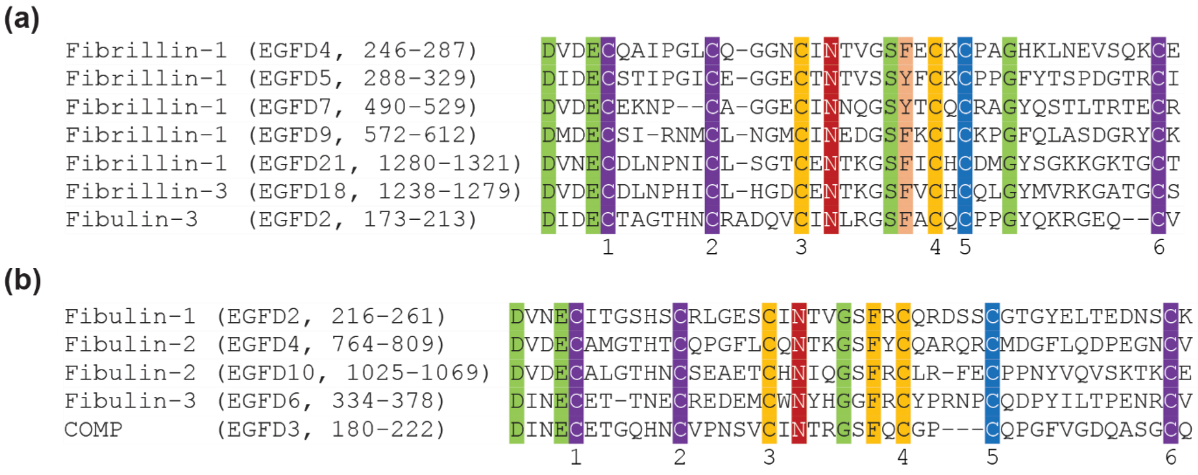

Cartilage oligomeric matrix protein (COMP)

Supporting Figure S3. Mass spectra of non-hydroxylated base peptides used to calculate the percentage site occupancy of hydroxylated asparagine in ovarian EGFDs which lack the C4-X-C5 motif. Analysis of a human ovary-derived proteome (1) reveals clear evidence for partial asparagine hydroxylation in EGFDs which lack the C4-X-C5 motif and which do not match the reported consensus EGFD requirements for AspH-catalyzed asparagine hydroxylation, *i.e.* C_3-X-D/N-X-X-X-X-F/Y-X-C_4-X-C_5 (2-7). A mixture of EGFD-derived peptide fragments with hydroxylated and non-hydroxylated asparagine residues was observed. The MS-MS spectra are shown for peptide fragments derived from ovarian (**a**) FBLN1 EGFD3, (**b**) FBLN5 EGFD1, and (**c**) HMCN1 EGFD2 which do not bear hydroxylated asparagine residues and which have been used as base peptides to calculate the percentage site occupancies of asparagine hydroxylation in ovarian EGFDs together with the corresponding hydroxylated peptide fragments shown in Figure 3.

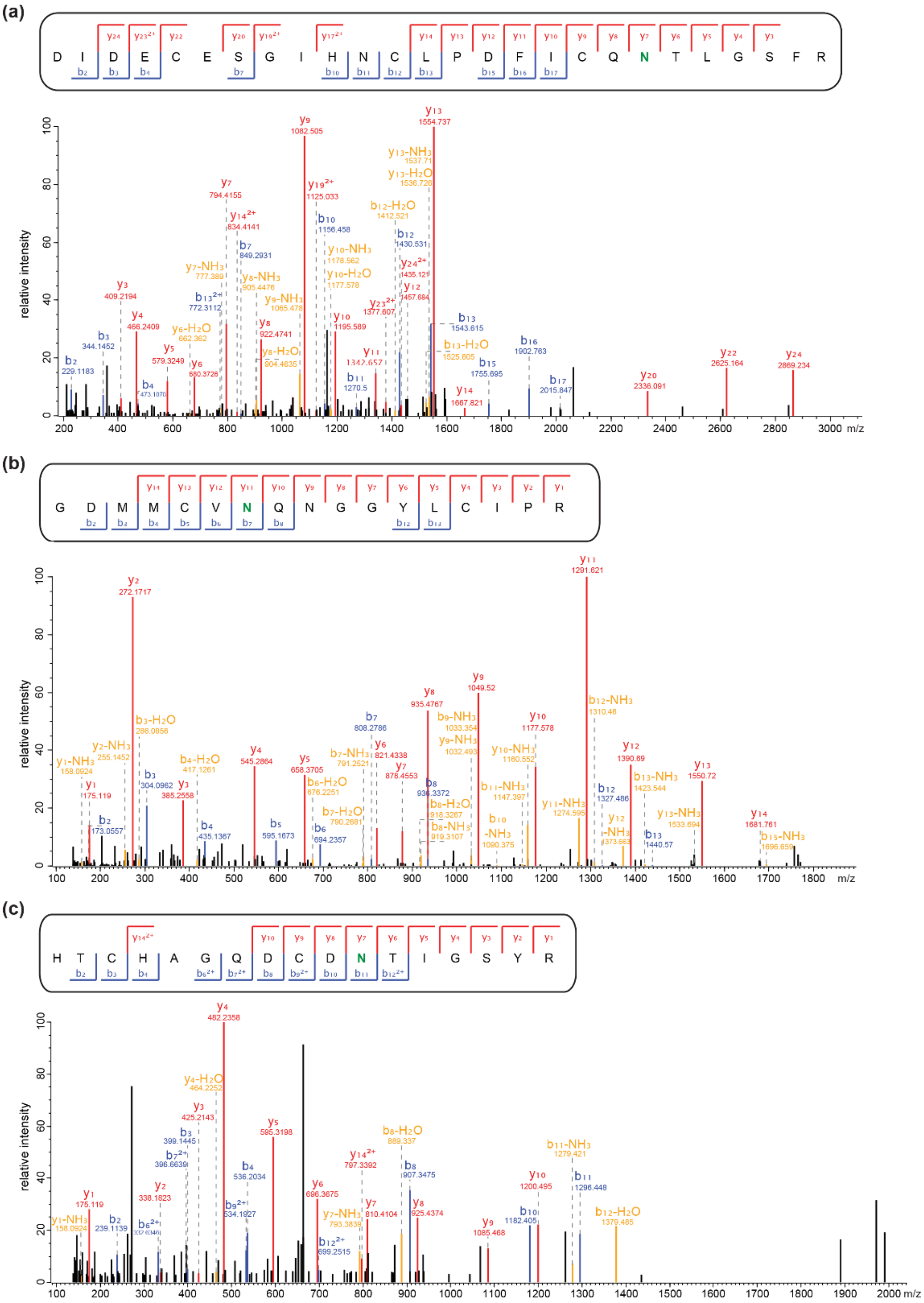

Supporting Figure S4. The thioether-bridged cyclic peptides used in this study (continues on the following three pages). The potential AspH substrate peptide sequences used based on the amino acid sequences of the indicated human proteins. Peptides were synthesized with C-terminal amides by solid phase peptide synthesis (SPPS), cyclized in a microwave reactor, and purified by HPLC as reported and described in the Experimental Procedures (9,10). A linear gradient (2−40%$_{v/v}$ over 40 min) of acetonitrile in water (each containing 0.1%$_{v/v}$ trifluoroacetic acid, TFA) was used as the eluent for HPLC purifications. The anticipated peptide masses and purity (>90% by HPLC) were confirmed using solid phase extraction coupled to mass spectrometry (SPE-MS) in buffer (50 mM HEPES, pH 7.5, 20 °C).

(**I**) (**a**) Sequence and purification characteristics of the human fibulin-1 EGFD3-derived cyclic peptide hFBLN1-CP$_{279\text{-}297}$ (**1**) used in this work. The predicted site for AspH-catalyzed hydroxylation is in red (N281), consensus sequence residues are in orange (F286, C288), and substituted residues are in light blue (D-alanine replacing C279 and serine replacing C294). The residue in green (P290) highlights the position at which a cysteine residue would be expected to form a C-X-C motif, based on the previous accepted consensus sequence for AspH-catalyzed EGFD hydroxylation (2-7). A thioether, obtained by reaction of the cysteine sulfur with an N-terminal *N*-chloroacetyl group, bridges residues ^{D}A279 and C288 forming a 10 amino acid membered macrocycle; (**b**) mass spectrum (SPE-MS) of **1** in reaction buffer (50 mM HEPES, pH 7.5). m/z = 1059.05 corresponds to the +2 charge state of **1**; the enlarged region shows the m/z +2 peak. m/z = 706.37 corresponds to the +3 charge state of **1**, m/z = 530.03 corresponds to the +4 charge state of **1**, and m/z = 239.11 corresponds to the +1 charge state of HEPES. The peptide is estimated >90% pure based on HPLC analysis. Note, m/z values referred to are for the most abundant isotope.

(a)

hFBLN1-CP$_{279\text{-}297}$ (**1**)

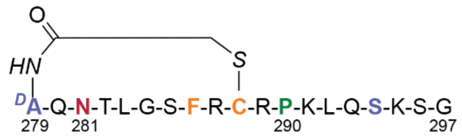

HPLC gradient: 2%$_{v/v}$ to 40%$_{v/v}$ MeCN/H_2O (+0.1%$_{v/v}$ TFA)
$\mathbf{t_R}$: 20.3 min

SPE-MS: m/z calculated for $C_{89}H_{151}N_{31}O_{27}S$ $[M+2H]^{2+}$: 1059.0553, found: 1059.0518

(b)

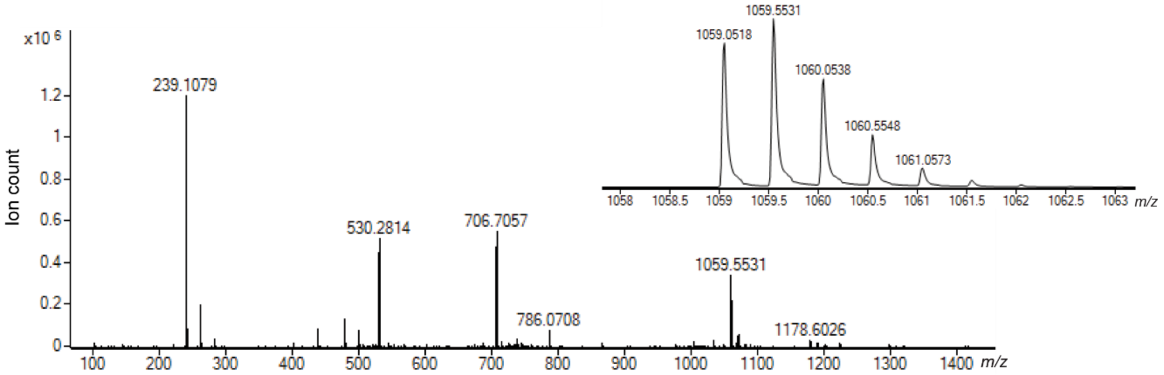

(**II**) (**a**) Sequence and purification characteristics of the human fibulin-5 EGFD1-derived cyclic peptide hFBLN5-$CP_{59\text{-}77}$ (**2**) used in this work. The predicted site for AspH-catalyzed hydroxylation is in red (N61), consensus sequence residues are in orange (Y66, C68), and D-alanine substituting for C59 is in light blue. The residue in green (P70) highlights the position at which a cysteine residue would be expected to form a C-X-C motif, based on the previous accepted consensus sequence for AspH-catalyzed EGFD hydroxylation (2-7). A thioether, obtained by reaction of the cysteine sulfur with an N-terminal *N*-chloroacetyl group, bridges residues ^{D}A59 and C68 forming a 10 amino acid membered macrocycle; (**b**) mass spectrum (SPE-MS) of **2** in reaction buffer (50 mM HEPES, pH 7.5). m/z = 1087.55 corresponds to the +2 charge state of **2**; the enlarged region shows the m/z +2 peak. m/z = 725.37 corresponds to the +3 charge state of **2**, and m/z = 239.11 corresponds to the +1 charge state of HEPES. The peptide is estimated >90% pure based on HPLC analysis. Note, m/z values referred to are for the most abundant isotope.

(a)

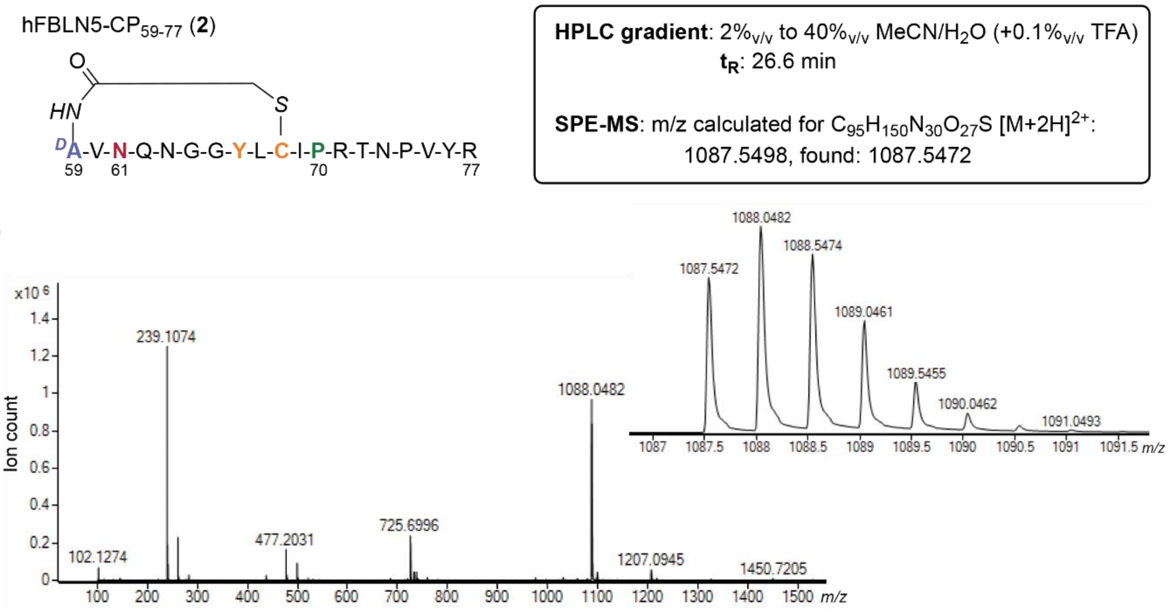

(**III**) (**a**) Sequence and purification characteristics of the human hemicentin-1 EGFD2-derived cyclic peptide hHMCN1-$CP_{5164\text{-}5182}$ (**3**) used in this work. The predicted site for AspH-catalyzed hydroxylation is in red (N5166), consensus sequence residues are in orange (Y5171, C5173), and substituted residues are in light blue (D-alanine replacing C5164 and serine replacing C5177). The residue in green (V5175) highlights the position at which a cysteine residue would be expected to form a C-X-C motif, based on the previous accepted consensus sequence for AspH-catalyzed EGFD hydroxylation (2-7). A thioether, obtained by reaction of the cysteine sulfur with an N-terminal *N*-chloroacetyl group, bridges residues ^{D}A5164 and C5173 forming a 10 amino acid membered macrocycle; (**b**) mass spectrum (SPE-MS) of **3** in reaction buffer (50 mM HEPES, pH 7.5). m/z = 1042.51 corresponds to the +2 charge state of **3**; the enlarged region shows the m/z +2 peak. m/z = 695.34 corresponds to the +3 charge state of **3**, and m/z = 239.11 corresponds to the +1 charge state of HEPES. The peptide is estimated >90% pure based on HPLC analysis. Note, m/z values referred to are for the most abundant isotope.

(a)

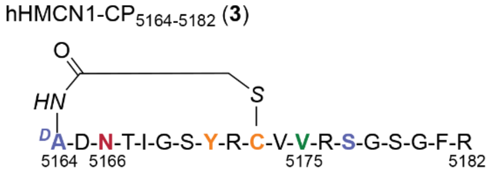

HPLC gradient: 2%$_{v/v}$ to 40%$_{v/v}$ MeCN/H_2O (+0.1%$_{v/v}$ TFA)
t$_R$: 22.3 min

SPE-MS: m/z calculated for $C_{87}H_{140}N_{30}O_{28}S$ $[M+2H]^{2+}$: 1042.5082, found: 1042.5039

(b)

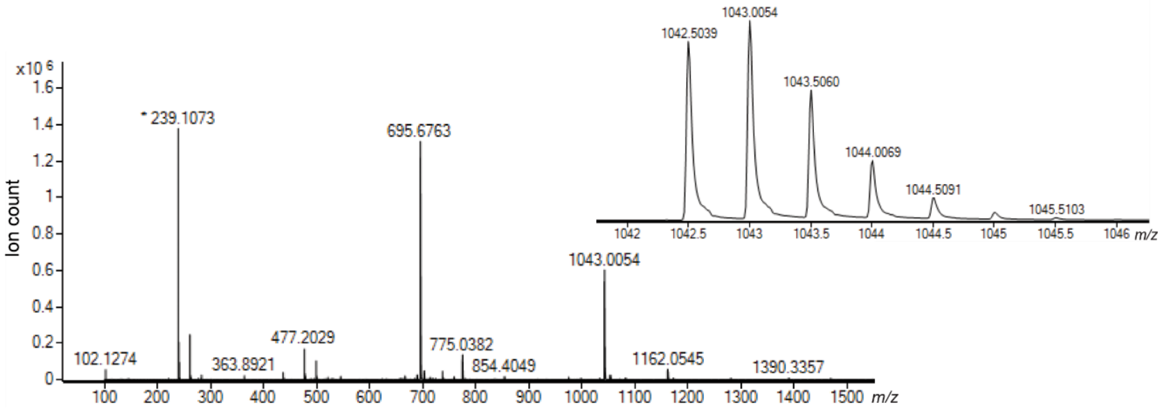

(**IV**) (**a**) Sequence and purification characteristics of the human lymphocyte antigen (Ly)-6/plasminogen activator urokinase receptor (PLAUR) domain (LU domain) containing protein 6B (LYPD6B)-derived cyclic peptide hLYPD6B-CP$_{65\text{-}83}$ (**4**) used in this work. The predicted site for AspH-catalyzed hydroxylation is in red (N67), consensus sequence residues are in orange (Y72, C74), and substituted residues are in light blue (D-alanine replacing C65 and serine replacing C83). The residue in green (R76) highlights the position at which a cysteine residue would be expected to form a C-X-C motif in an EGFD, based on the previous accepted consensus sequence for AspH-catalyzed EGFD hydroxylation (2-7). A thioether, obtained by reaction of the cysteine sulfur with an N-terminal *N*-chloroacetyl group, bridges residues ^{D}A65 and C74 forming a 10 amino acid membered macrocycle; (**b**) mass spectrum (SPE-MS) of **4** in reaction buffer (50 mM HEPES, pH 7.5). m/z = 1141.44 corresponds to the +2 charge state of **4**; the enlarged region shows the m/z +2 peak. m/z = 239.11 corresponds to the +1 charge state of HEPES. The peptide is estimated >90% pure based on HPLC analysis. Note, m/z values referred to are for the most abundant isotope.

(a)

hLYPD6B-CP$_{65\text{-}83}$ (**4**)

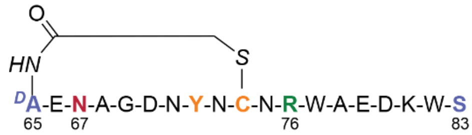

HPLC gradient: 2%$_{v/v}$ to 40%$_{v/v}$ MeCN/H_2O (+0.1%$_{v/v}$ TFA)
t_R: 19.6 min

SPE-MS: m/z calculated for $C_{96}H_{134}N_{30}O_{34}S$ $[M+2H]^{2+}$: 1141.4694, found: 1141.4414

(b)

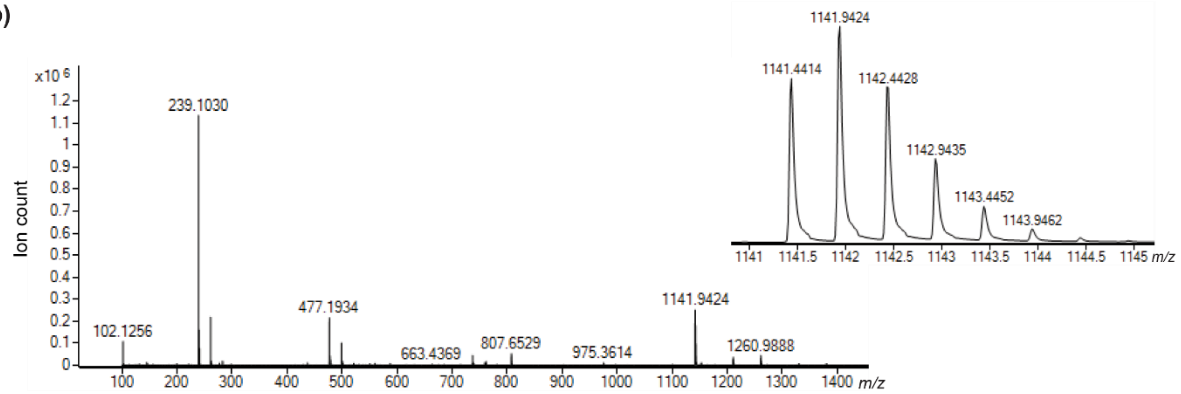

Supporting Figure S5. LYPD6B could be an AspH substrate based on sequence and structure analyses. (**a**) Sequence analysis of human lymphocyte antigen (Ly)-6/plasminogen activator urokinase receptor (PLAUR) domain (LU domain) containing protein 6B (LYPD6B; UniProt ID: Q8NI32) reveals that LYPD6B contains a motif that apparently fits the revised sequence requirements for AspH catalysis, *i.e.* a C-X-D/N-X-X-X-X-F/Y-X-C motif (Figure 7); N67 could potentially be (partially) hydroxylated by AspH. The potential hydroxylation site is in red (N67); consensus sequence residues required for AspH catalysis are in orange and salmon; other cysteine residues are in purple; (**b**) a view of the LYPD6B fold determined by NMR (LYPD6B residues 60-154; PDB ID: 6ZSO (11)) highlighting its disulfide connectivity pattern; (**c**) the potential hydroxylation site, *i.e.* N67, is buried in a disulfide-bridged 10 amino acid residue-membered macrocycle (C65 to C74 as highlighted in orange) on the LYPD6B surface, rendering it, at least potentially, accessible for AspH (PDB ID: 6ZSO (11)).

(a)

```
                   10         20         30         40         50         60
LYPD6B   MLYKSSDRPA HKVSMLLLCH ALAIAVVQIV IFSESWAFAK NINFYNVRPP LDPTPFPNSF

                   70         80         90        100        110        120
LYPD6B   KCFTCENAGD NYNCNRWAED KWCPQNTQYC LTVHHFTSHG RSTSITKKCA SRSECHFVGC

                  130        140        150        160        170        180
LYPD6B   HHSRDSEHTE CRSCCEGMIC NVELPTNHTN AVFAVMHAQR TSGSSAPTLY LPVLAWVFVL PLL
```

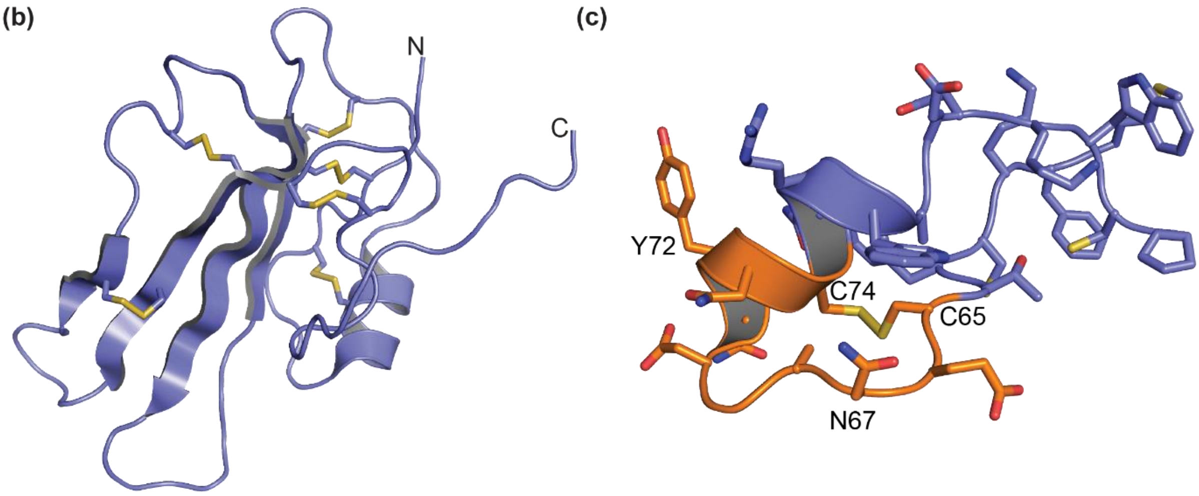

Supporting Figure S6. Hydroxylation rates of AspH-catalyzed hydroxylations of the synthetic hemicentin-derived peptide hHNCT1-CP$_{5164\text{-}5182}$ used to determine kinetic parameters. The maximum velocity (v_{max}) and the Michaelis constant (K_m) of AspH for hHMCN1-CP$_{5164\text{-}5182}$ (Experimental Procedures) were determined in independent triplicates, monitoring AspH-catalyzed hydroxylation of hHNCT1-CP$_{5164\text{-}5182}$ by SPE-MS as described (Experimental Procedures). Conditions: 0.1 µM His$_6$-AspH$_{315\text{-}758}$, 100 µM L-ascorbic acid (LAA), 20 µM 2OG, 20 µM ammonium iron(II) sulfate hexahydrate (FAS, $(NH_4)_2Fe(SO_4)_2 \cdot 6H_2O$), and variable concentrations of the hHNCT1-CP$_{5164\text{-}5182}$ peptide in buffer (50 mM HEPES, pH 7.5, 20 °C). Measurement times were normalized to the first sample injection analyzed after the addition of AspH to the Substrate Mixture (t = 0 s), by which time low levels of hydroxylation were manifest. Data are shown as mean of three independent runs (n = 3; mean ± standard deviation, SD).

(**a**) Time course of the AspH-catalyzed hydroxylation reaction for the shown concentrations of hHMCN1-CP$_{5164\text{-}5182}$ peptide; and (**b**) hydroxylation rates used to determine kinetic parameters of AspH for hHMCN1-CP$_{5164\text{-}5182}$ peptide.

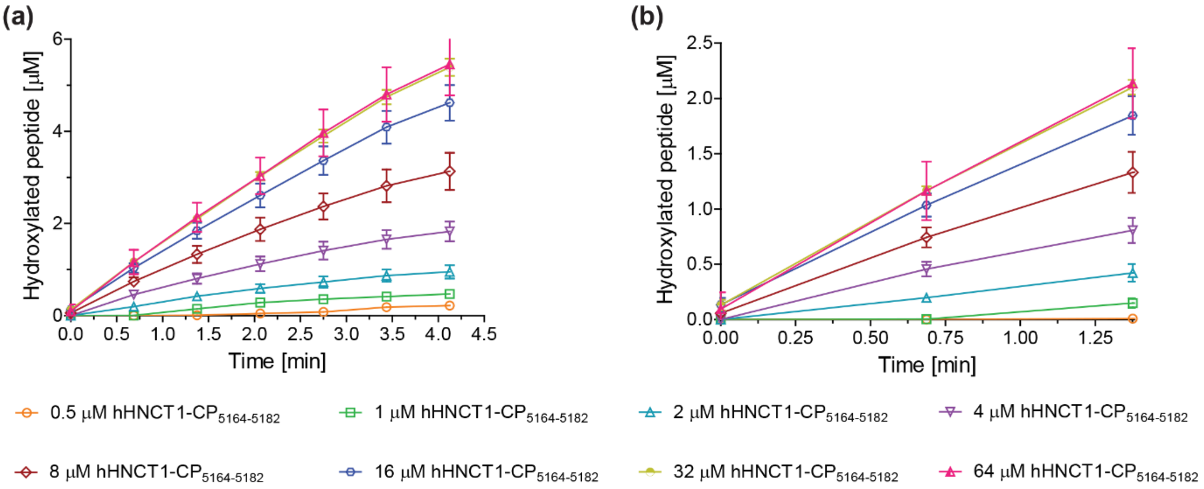

Supporting Figure S7. Hydroxylation rates of AspH-catalyzed hydroxylations of the synthetic Ly6/PLAUR domain-containing protein 6B (LYPD6B)-derived peptide hLYPD6B-CP$_{65\text{-}83}$ used to determine kinetic parameters. The maximum velocity (v_{max}) and the Michaelis constant (K_m) of AspH for hHNCT1-CP$_{5164\text{-}5182}$ (Experimental Procedures) were determined in independent triplicates, monitoring AspH-catalyzed hydroxylation of hLYPD6B-CP$_{65\text{-}83}$ by SPE-MS as described (Experimental Procedures). Conditions: 0.1 μM His$_6$-AspH$_{315\text{-}758}$, 100 μM LAA, 20 μM 2OG, 20 μM FAS ($(NH_4)_2Fe(SO_4)_2 \cdot 6H_2O$), and variable concentrations of the hLYPD6B-CP$_{65\text{-}83}$ peptide in buffer (50 mM HEPES, pH 7.5, 20 °C). Measurement times were normalized to the first sample injection analyzed after the addition of AspH to the Substrate Mixture (t = 0 s), by which time low levels of hydroxylation were manifest. Data are shown as mean of three independent runs (n = 3; mean ± SD).
(**a**) Time course of the AspH-catalyzed hydroxylation reaction for the shown concentrations of hLYPD6B-CP$_{65\text{-}83}$ peptide; and (**b**) hydroxylation rates used to determine kinetic parameters of AspH for hLYPD6B-CP$_{65\text{-}83}$ peptide.

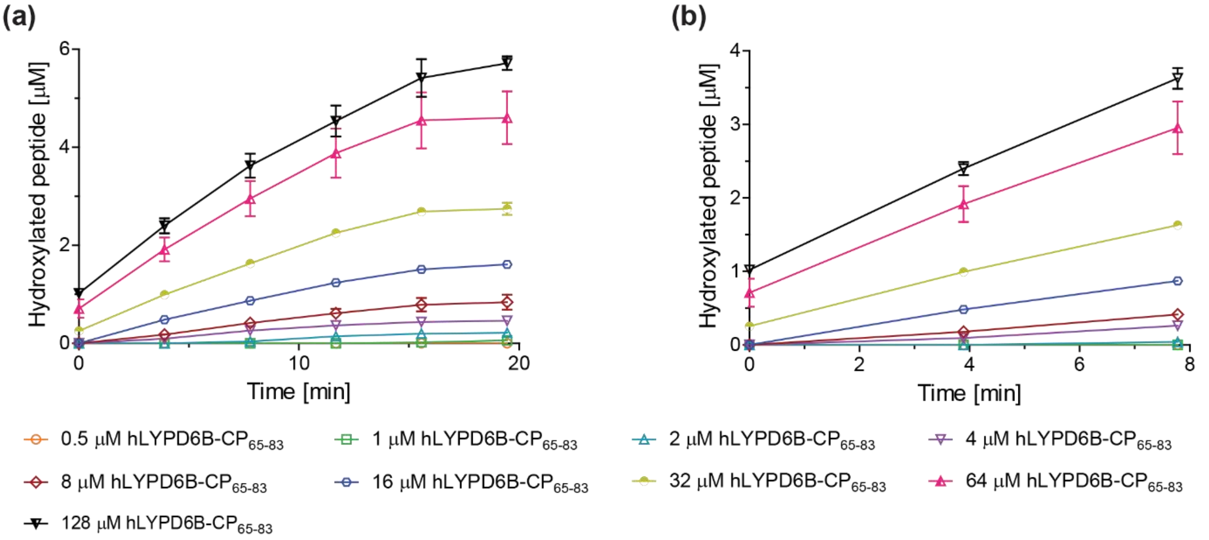

References

1. Wang, D., Eraslan, B., Wieland, T., Hallström, B., Hopf, T., Zolg, D. P., Zecha, J., Asplund, A., Li, L.-h., Meng, C., Frejno, M., Schmidt, T., Schnatbaum, K., Wilhelm, M., Ponten, F., Uhlen, M., Gagneur, J., Hahne, H., and Kuster, B. (2019) A deep proteome and transcriptome abundance atlas of 29 healthy human tissues. *Mol. Syst. Biol.* **15**, e8503
2. Stenflo, J., Lundwall, Å., and Dahlbäck, B. (1987) β-Hydroxyasparagine in domains homologous to the epidermal growth factor precursor in vitamin K-dependent protein S. *Proc. Natl. Acad. Sci. USA* **84**, 368-372
3. Stenflo, J., Ohlin, A. K., Owen, W. G., and Schneider, W. J. (1988) β-Hydroxyaspartic acid or β-hydroxyasparagine in bovine low density lipoprotein receptor and in bovine thrombomodulin. *J. Biol. Chem.* **263**, 21-24
4. Greve, J. M., Pinkham, A. M., and Cowan, J. A. (2021) Human aspartyl (asparaginyl) hydroxylase. A multifaceted enzyme with broad intra- and extra-cellular activity. *Metallomics* **13**, mfab044
5. Stenflo, J. (1991) Structure-function relationships of epidermal growth factor modules in vitamin K-dependent clotting factors. *Blood* **78**, 1637-1651
6. Siggs, O. M., Souzeau, E., and Craig, J. E. (2019) Loss of ciliary zonule protein hydroxylation and lens stability as a predicted consequence of biallelic ASPH variation. *Ophthalmic Genet.* **40**, 12-16
7. Stenflo, J., Holme, E., Lindstedt, S., Chandramouli, N., Tsai Huang, L. H., Tam, J. P., and Merrifield, R. B. (1989) Hydroxylation of aspartic acid in domains homologous to the epidermal growth factor precursor is catalyzed by a 2-oxoglutarate-dependent dioxygenase. *Proc. Natl. Acad. Sci. USA* **86**, 444-447
8. Millikin, R. J., Solntsev, S. K., Shortreed, M. R., and Smith, L. M. (2018) Ultrafast peptide label-free quantification with FlashLFQ. *J. Proteome Res.* **17**, 386-391
9. Pfeffer, I., Brewitz, L., Krojer, T., Jensen, S. A., Kochan, G. T., Kershaw, N. J., Hewitson, K. S., McNeill, L. A., Kramer, H., Münzel, M., Hopkinson, R. J., Oppermann, U., Handford, P. A., McDonough, M. A., and Schofield, C. J. (2019) Aspartate/asparagine-β-hydroxylase crystal structures reveal an unexpected epidermal growth factor-like domain substrate disulfide pattern. *Nat. Commun.* **10**, 4910
10. Brewitz, L., Tumber, A., and Schofield, C. J. (2020) Kinetic parameters of human aspartate/asparagine-β-hydroxylase suggest that it has a possible function in oxygen sensing. *J. Biol. Chem.* **295**, 7826-7838
11. Paramonov, A. S., Kocharovskaya, M. V., Tsarev, A. V., Kulbatskii, D. S., Loktyushov, E. V., Shulepko, M. A., Kirpichnikov, M. P., Lyukmanova, E. N., and Shenkarev, Z. O. (2020) Structural diversity and dynamics of human three-finger proteins acting on nicotinic acetylcholine receptors. *Int. J. Mol. Sci.* **21**, 7280
